# Targeting the E2F6-TOP2A-DKK1 axis: a novel therapeutic strategy for EMT-driven hepatocellular carcinoma progression

**DOI:** 10.3389/fimmu.2026.1809952

**Published:** 2026-07-02

**Authors:** Mindan Xing, Yi Lu, Yan Guo, Yantao Jiang, Hengqi Liu, Junjie Yu, Luyao Tong, Zhongyu Wang, Chao Li, Xing Chen, Wei Luo

**Affiliations:** 1 The First Clinical Medical College of Shanxi Medical University, Taiyuan, Shanxi, China; 2 Department of Gastroenterology, First Hospital of Shanxi Medical University, Taiyuan, Shanxi, China; 3 Tianjin Medical University Cancer Institute and Hospital, National Clinical Research Center for Cancer; Key Laboratory of Cancer Prevention and Therapy; Tianjin’s Clinical Research Center for Cancer; Tianjin Lung Cancer Center, Tianjin Cancer Institute & Hospital, Tianjin Medical University, Tianjin, China; 4 Hepatobiliary Surgery, Zhuji Affiliated Hospital of Wenzhou Medical University, Zhuji, Zhejiang, China

**Keywords:** DKK1, E2F6, epithelial–mesenchymal transition, hepatocellular carcinoma, TOP2A

## Abstract

**Background:**

Hepatocellular carcinoma (HCC) has a poor prognosis, and identifying key driver genes and their molecular mechanisms is crucial for improving patient outcomes. While TOP2A is dysregulated in multiple cancers, its role and regulatory network in HCC remain incompletely understood.

**Methods:**

Integrated bioinformatics analyses were performed using TCGA, ICGC, and GEO datasets. TOP2A expression and prognostic value were validated in 120 clinical samples via immunohistochemistry. Functional assays (including CCK-8, Transwell, and wound healing *in vitro*, as well as xenograft tumor models *in vivo*) were conducted to assess phenotypic effects. Mechanisms were explored using ChIP-PCR, WB, and RT-qPCR. Prognostic models were developed using multivariate regression and evaluated using calibration curves, ROC, and DCA.

**Results:**

TOP2A was significantly upregulated in HCC tissues, with an AUC of 0.935 for diagnosis, and high expression correlated with advanced stage and poor survival (p<0.05). A prognostic model incorporating TOP2A expression, TNM stage, and tumor grade showed robust predictive accuracy for 1-, 3-, and 5-year survival (AUCs: 0.77, 0.85, 0.75). Knockdown of TOP2A suppressed proliferation, migration, and invasion. Mechanistically, E2F6 transcriptionally activated TOP2A by binding to its promoter, while TOP2A promoted EMT by upregulating DKK1 and activating β-catenin signaling. High TOP2A expression predicted poor response to TACE, sorafenib, and immunotherapy. The candidate targeted agent, A-443654, alone or combined with anti-PD-L1, potently suppressed tumor growth *in vivo*.

**Conclusion:**

This study delineates the E2F6-TOP2A-DKK1 axis as a key mechanism in HCC progression. TOP2A serves as a diagnostic and prognostic biomarker, and targeting this pathway offers a promising therapeutic strategy for HCC.

## 1 Introduction

Liver cancer ranks as the sixth most commonly diagnosed cancer and the third leading cause of cancer-related mortality worldwide (1). Hepatocellular carcinoma (HCC), the predominant form of primary liver cancer, represents a formidable global health burden (2). Despite advancements in diagnosis and therapy, the prognosis for HCC patients remains poor, with a five-year survival rate below 20% (3). This dismal outcome is largely attributed to the high heterogeneity of HCC, which confounds early detection, limits treatment efficacy, and underscores the urgent need for a deeper understanding of its molecular drivers and the development of personalized therapeutic strategies. In this pursuit, DNA topoisomerases have garnered significant attention for their crucial roles in cancer biology.

DNA topoisomerases are essential enzymes that resolve topological constraints during DNA replication and transcription. Among them, DNA topoisomerase IIα (TOP2A) (4), encoded by the TOP2A gene on chromosome 17q12-21, is a cell cycle-dependent enzyme crucial for cell proliferation, with peak expression during the G2/M phase (5, 6). Beyond its physiological role, TOP2A has emerged as a significant player in oncogenesis. Alterations in TOP2A, including copy number variations (7), somatic mutations (8, 9), and aberrant overexpression (10–12), are frequently observed in various cancers and are often associated with aggressive tumor behavior and poor survival.

This oncogenic role of TOP2A is also evident in HCC. Elevated TOP2A expression is linked to early disease occurrence, reduced survival, and chemoresistance (11, 13–15). Mechanistically, TOP2A has been shown to promote HCC cell migration by upregulating the transcription factor Snail, thereby suppressing E-cadherin and driving epithelial–mesenchymal transition (EMT), likely via the p-ERK1/2/p-SMAD2/Snail pathways (13). Furthermore, TOP2A transcription is regulated by specific factors through distinct mechanisms. The transcription factor AP-2α directly binds to the TOP2A promoter (16), while the epigenetic modifier WHSC1 increases its expression by inducing histone H3 lysine 36 dimethylation (H3K36me2) at the promoter region (17). While the oncogenic role of TOP2A in HCC is recognized, the core circuitry governing its expression and function—encompassing its direct regulation by E2F6, its activation of the DKK1/β-catenin axis to drive EMT, and the clinical translatability of this pathway—remains largely unexplored.

In this study, we aimed to address these gaps. Our findings demonstrated that E2F6 is a novel transcriptional factor of TOP2A and elucidated the TOP2A-DKK1-β-catenin axis as a key mechanistic driver of EMT and metastasis in HCC. Furthermore, we translated these insights into clinically applicable tools, developing molecular-based diagnostic and prognostic models, and proposing a novel therapeutic strategy combining a candidate TOP2A-targeting agent with immune checkpoint blockade.

## 2 Materials and methods

### 2.1 Data collection

Expression profile datasets for HCC, GSE109211 (18) and GSE104580, were obtained from the Gene Expression Omnibus (GEO) database (https://www.ncbi.nlm.nih.gov/geo/). Both datasets consisted of samples derived from homo sapiens. GSE109211 included data from 140 HCC cases (18), while GSE104580 comprised 147 HCC cases, all of which were analyzed in this study (**Supplementary Table 1**).

Using the TCGAbiolinks R package (version 2.32.0) (19), we retrieved FPKM expression profile data, count data matrix, survival data, and clinical data for 371 patients with liver hepatocellular carcinoma (LIHC) from The Cancer Genome Atlas (TCGA) database. Among these, expression profile data for 371 tumor tissues and 50 non-tumor tissues were used for differential expression analysis. Patients with incomplete clinical or survival data were excluded, resulting in the inclusion of data from 337 patients for survival and prognosis analyses. Count data, survival data, and clinical information for 231 HCC patients were obtained from the legacy version of the International Cancer Genome Consortium (ICGC) database (20) for further analysis (**Supplementary Table 1**). The ICGC Liver Cancer (LIRI-JP) data were accessed through the ICGC Data Portal (https://dcc.icgc.org/projects/LIRI-JP) during its open-access period (2014.01-2023.01). As a legacy public dataset at the time of acquisition, these data were available under the ICGC Open Data Policy (https://icgc.org/policies/) without requiring project-specific approval (21).

**Figure 1:**
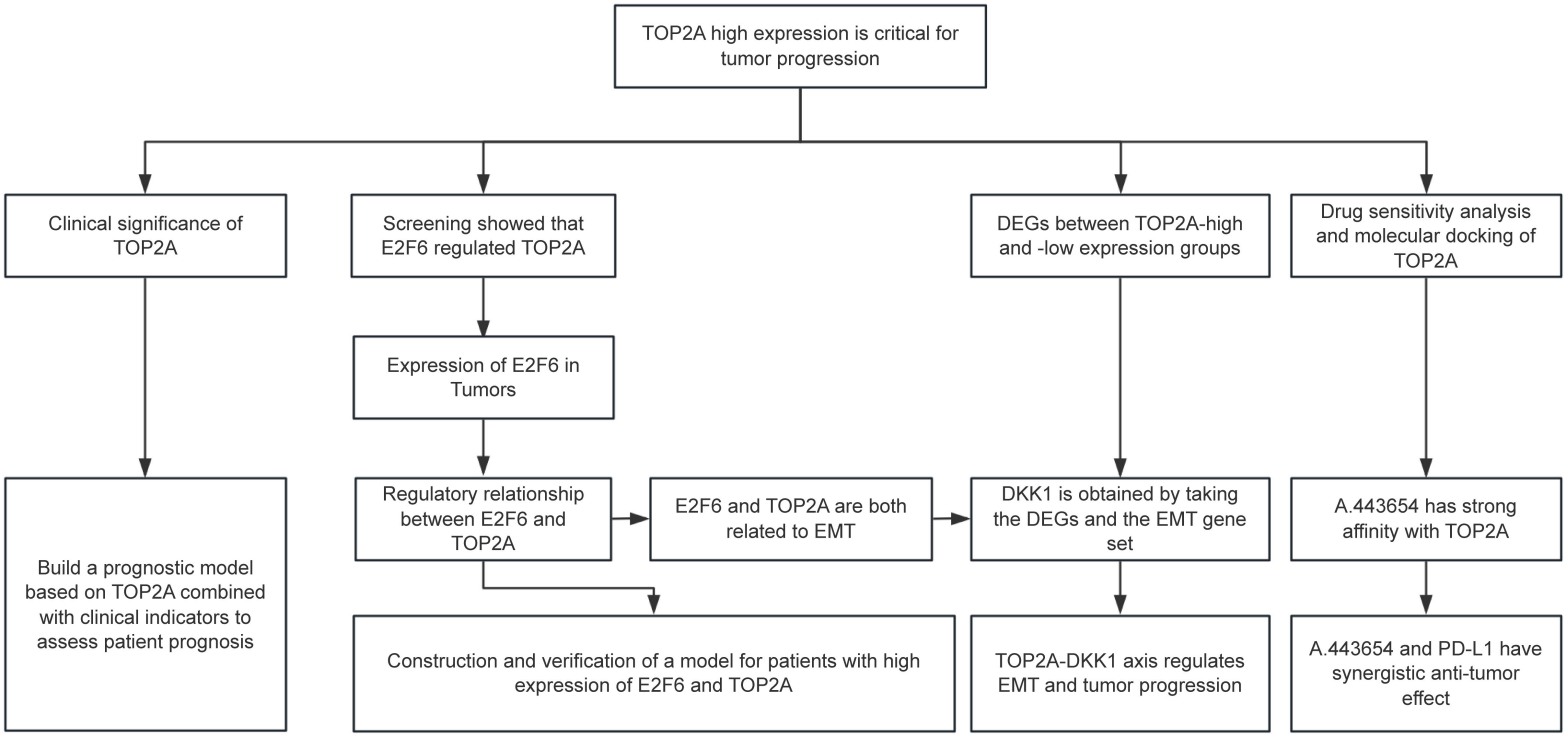
The workflow of this study.

Xiantao Academic (https://www.xiantaozi.com/) was used to analyze the expression of TOP2A and E2F6 across 33 tumor types in the TCGA database. The UALCAN database (https://ualcan.path.uab.edu) was employed to assess the protein expression of TOP2A in HCC (22, 23). Transcription factors associated with TOP2A were investigated using the LncMAP web tool (https://bio-bigdata.hrbmu.edu.cn/LncMAP/) (24). The UCSC Genome Browser (https://genome.ucsc.edu/) (25) and JASPAR database (26) (https://jaspar.elixir.no/) were leveraged to predict transcription factors regulating TOP2A. **Figure 1** illustrates the workflow of this study.

### 2.2 Cell lines and cell culture

The normal human hepatocyte cell line HL-7702, human hepatocellular carcinoma cell lines (Huh7, PLC/PRF/5, HCCLM3, HepG2, SNU387), Murine hepatoma cell line Hepa 1–6 and the HEK293T cell line were purchased from the American Type Culture Collection (ATCC, USA). All cell lines were cultured in high-glucose Dulbecco’s Modified Eagle Medium (DMEM) or RPMI-1640 medium (Gibco, USA), supplemented with 10% fetal bovine serum (FBS; Gibco, USA) and 1% penicillin-streptomycin (HyClone, USA). All cultures were maintained at 37 °C in a humidified incubator with 5% CO_2_.

### 2.3 Lentivirus production and generation of stable cell lines

Lentiviruses for gene knockdown (TOP2A shRNA1, TOP2A shRNA2, E2F6 shRNA1, E2F6 shRNA2) were purchased from Shanghai Genesci Medical Technology Co., Ltd., and DKK1 shRNA was obtained from GenePharma (Shanghai, China). A TOP2A overexpression construct was generated by subcloning its coding sequence (NCBI Reference Sequence: NM_001067.4) into the pcDNA3.1 vector (Invitrogen).

According to the manufacturer’s instructions, lentiviral vectors were co-transfected into HEK293T cells along with the packaging plasmids psPAX2 (Addgene) and pMD2.G (Addgene) using Lipofectamine 2000 transfection reagent (Invitrogen, Shanghai, China). The lentivirus-containing supernatant was collected 48 hours post-transfection, filtered through a 0.22 μm membrane (Millipore), and subsequently used to infect Huh7, HepG2 and HCCLM3 cells. Stable cell lines were selected by adding 1 μg/ml puromycin to the culture medium. The shRNA sequences were as follows:

TOP2A shRNA1: 5′-CCCAACTTTGATGTGCGTGAA-3′;

TOP2A shRNA2: 5′-GCTCCAAATCAATATGTGATT-3′;

DKK1 shRNA: 5′-GATCCGTACCAAGCATAGGAGAAATTCAAGAGATTTCTCCTATGCTTGGTACTTTTTTG-3′;

E2F6 shRNA1: 5′-CCGGATAGATGTACACACGAATTTACTCGAGT-3′;

E2F6 shRNA2: 5′-CCGGTTGATGTATCGCTGGTTTATTCTCGA-3′.

### 2.4 Differential expression analysis

Differential expression analysis was conducted using three widely adopted statistical methods: edgeR (27), limma (28), and DESeq2 (29). Raw RNA-seq data underwent quality control processing, followed by cross-sample normalization of gene expression counts. Differentially expressed genes (DEGs) were identified by applying a threshold of |log_2_ fold change (log_2_FC)| > 2 and adjusted p < 0.05 for the comparison between patients with high co-expression of E2F6 and TOP2A and other patients. Specifically, for comparisons between TOP2A-high and TOP2A-low expression groups, the threshold was set at |log_2_FC| > 1.5 and adjusted p < 0.05.

### 2.5 Construction and validation of the predictive model

To accurately identify HCC with high expression both of E2F6 and TOP2A (E2F6_H&TOP2A_H), a predictive model was developed using logistic regression analysis based on the expression levels of E2F6 and TOP2A in the TCGA-LIHC dataset. Predictive scores for each tumor sample were calculated using the model, with the formula for the predictive score defined as follows:


$$P\text{redictScore=}\sum_{\text{i=l}}^{\text{N}}\text{X}{\times \text{Y}}_{i}$$

X represents the coefficients of the multifactor logistic regression analysis, and Y denotes the expression level of the gene. The regplot R package (version 1.1) was used to construct a nomogram, comparing predicted values with observed values to generate a calibration curve for assessing the nomogram’s performance. Receiver operating characteristic (ROC) curve analysis was performed using MedCalc software to evaluate the predictive accuracy of the score for identifying E2F6_H&TOP2A_H patients. The ggDCA R package (version 1.2) was employed for decision curve analysis (DCA) to assess the clinical utility of the model. To validate the model’s accuracy and stability, datasets from ICGC-HCC, GSE109211, and GSE104580 were analyzed using both ROC and DCA methods.

### 2.6 Functional enrichment analysis

Gene Ontology (GO) enrichment analysis provides comprehensive functional annotations of genes in three categories: biological processes (BP), molecular functions (MF), and cellular components (CC) (30). Kyoto Encyclopedia of Genes and Genomes (KEGG) pathway analysis identifies biological pathway alterations caused by gene changes across experimental groups (31–33). The enrichGO function of the clusterProfiler R package (version 4.12.6) was used to perform GO and KEGG enrichment analyses, and results were visualized to highlight the significantly enriched GO terms and pathways.

Single-sample gene set enrichment analysis (ssGSEA) normalizes and ranks gene expression values in a given sample, generating enrichment scores based on specific gene sets and the cumulative distribution function of remaining genes (34). The ssgsea function in the GSVA R package (version 1.42.0) was employed to score phenotypes, pathways, immune cells, and immune-related pathways, assessing differences between HCC subgroups.

Gene Set Enrichment Analysis (GSEA) (35) was conducted with the clusterProfiler R package (36) to detect enriched biological pathways across all protein-coding genes. Genes were ranked by their log_2_ fold change values derived from limma-based differential expression analysis. Enrichment was evaluated against the hallmark gene sets from the Molecular Signatures Database (MSigDB; https://www.gsea-msigdb.org). A normalized enrichment score (NES) was calculated for each gene set to quantify its enrichment within the ranked gene list. Gene sets with |NES| > 1 and a false discovery rate (FDR) < 0.05 were deemed statistically significant.

### 2.7 Western blotting

Proteins were extracted from cells or tissues using RIPA lysis buffer (R0020, Solarbio, China), and their concentrations were determined with a BCA assay kit (PC0020, Solarbio). The protein samples were then denatured by boiling in loading buffer (LT 101, Epizyme, China). Following separation by SDS-PAGE, the proteins were transferred to a PVDF membrane using a standard wet transfer apparatus (BIO-RAD, WJ002, Epizyme). The membrane was blocked with 5% skim milk (D8304, Solarbio) and subsequently incubated overnight at 4 °C with the indicated primary antibodies. After incubation with the corresponding HRP-conjugated secondary antibodies (1:5000; Beyotime, A0208 and A0216), protein signals were visualized using an Omni‐ECL Femto chemiluminescence kit (SQ201, Solarbio). The primary antibodies used in this study were as follows: anti-TOP2A (Proteintech, 20233-1-AP, 1:2000), anti-E2F6 (Proteintech, 10691-1-AP, 1:1000), anti-DKK1 (Proteintech, 21112-1-AP, 1:2000), anti-N-cadherin (ABclonal, A0433, 1:1000), anti-E-cadherin (Proteintech, 20874-1-AP, 1:20000), anti-β-catenin (Proteintech, 51067-2-AP, 1:5000), anti-Snail (ABclonal, A11794, 1:1000), and anti-GAPDH (Proteintech, 10494-1-AP, 1:5000).

### 2.8 RNA extraction and real-time quantitative PCR

Total RNA was isolated from hepatocellular carcinoma cells using TRIzol reagent (G3013, Servicebio, China). Complementary DNA (cDNA) was synthesized from the extracted RNA using the HiScript II First Strand cDNA Synthesis Kit (R212-01, Vazyme, China). Quantitative real-time PCR (qPCR) was subsequently performed with the HiScript II Q RT SuperMix for qPCR (R223-01, Vazyme, China) and gene-specific primers, following the manufacturer’s protocol. The relative mRNA expression levels were calculated by the 2−ΔΔCt method, with GAPDH serving as the internal control. The sequences of all primers used are listed in **Supplementary Table 2**.

### 2.9 CCK8 assay and colony formation assay

For the CCK-8 assay, 2,000 Huh7, HepG2 or HCCLM3 cells stably expressing shRNA were seeded per well in 96-well plates. After a 2-hour incubation with CCK-8 reagent, the absorbance at 450 nm was measured using a spectrophotometer. For the colony formation assay, 1,000 infected cells were plated in 10 cm culture dishes with 10 mL of complete medium and cultured for two weeks. The resulting colonies were fixed and stained with 0.1% crystal violet (in methanol) for 30 minutes, after which the number of colonies was counted and documented. All experiments were performed with three independent biological replicates per group and repeated in triplicate.

### 2.10 Cell migration and invasion assays

Cell migration was assessed using a scratch wound assay. Briefly, Huh7, HepG2 and HCCLM3 cells were seeded in 6-well plates and transduced with lentiviral shRNA. Upon reaching 90–100% confluence, a uniform wound was created in each monolayer using a sterile 10 μL pipette tip. After washing, the cells were cultured in serum-free medium, and wound closure was monitored by imaging at 0 and 24 hours. Migration was further evaluated using a Transwell assay without Matrigel coating. For the invasion assay, Transwell filters were pre-coated with Matrigel (30 µL per insert). In both assays, 1×10^5^ lentivirus-transduced cells in 200 μL serum-free medium were plated in the upper chamber, while the lower chamber contained 600 μL of complete medium with 10% FBS as a chemoattractant. After 24 hours of incubation, the cells that migrated or invaded to the lower surface were fixed with 4% paraformaldehyde, stained with 0.1% crystal violet, and imaged. Cell numbers were quantified by counting five random fields per insert. All experiments were performed in triplicate with three independent biological replicates.

### 2.11 Immunohistochemistry

This study utilized a clinical cohort of 120 surgical liver cancer specimens (collected between November 2021 and January 2023) from the First Hospital of Shanxi Medical University, with approval from the institutional Ethics Committee (KYYJ-2023-071) and in accordance with the Declaration of Helsinki. All patients provided informed consent, and clinical follow-up extended to May 20, 2025. Tumor tissue specimens were fixed in formalin and embedded in paraffin. Sections were then deparaffinized in xylene and rehydrated through a graded ethanol series. Antigen retrieval was performed using a microwave heating method in an appropriate buffer. Endogenous peroxidase activity was quenched by incubation with 3% hydrogen peroxide. The sections were incubated overnight at 4 °C with a primary antibody against TOP2A (1:400 dilution). After washing with PBS, the sections were treated with an antibody signal enhancer and subsequently incubated with an HRP-conjugated secondary antibody. Immunoreactivity was visualized using a DAB chromogen substrate, and the sections were counterstained with hematoxylin. Staining intensity was quantitatively analyzed using Image Pro Plus software (version 6.0) for image standardization and preliminary assessment. Subsequently, immunohistochemical staining was independently evaluated by two blinded pathologists. Staining intensity was scored as 0 (negative), 1 (weak), 2 (moderate), or 3 (strong), and the percentage of positive cells was scored as 0 (<5%), 1 (5–25%), 2 (26–50%), 3 (51–75%), and 4 (>75%). The final immunoreactivity score was calculated by multiplying intensity and percentage scores. Discrepancies were resolved by joint re-evaluation. Samples with missing or incomplete data were excluded from the corresponding analyses. No imputation was performed.

### 2.12 Chromatin immunoprecipitation assays

The ChIP assay was conducted following the instructions for the SimpleChIP Enzymatic Chromatin IP Kit (Cell Signaling Technology, #9003). In brief, cells were fixed with 1% formaldehyde for 10 min, and cross-linking was quenched with glycine. Isolated nuclei were subjected to enzymatic shearing using micrococcal nuclease (20 min, 37 °C), and the reaction was stopped with EDTA. Complete nuclear lysis was achieved by sonication in ChIP buffer (3 cycles of 30-s pulses/30-s pauses). Sheared chromatin was immunoprecipitated by incubation with 10 μg of antibody overnight at 4 °C with rotation. Complexes were captured with magnetic beads (2 h, 4 °C), washed with low- and high-salt buffers, and the DNA was eluted. Precipitated DNA was quantified by qPCR using primers listed in **Supplementary Table 3**.

### 2.13 Immunofluorescence

To evaluate the effect of altered DKK1 expression on the intracellular localization of β-catenin, β-catenin immunofluorescence (IF) staining was performed in both experimental and control groups. Briefly, collected cells were fixed with 4% paraformaldehyde for 20 min and washed three times with phosphate-buffered saline (PBS). The cells were then permeabilized with 0.2% Triton X-100 for 15 min at room temperature, followed by three washes with PBS. Subsequently, cells were blocked with 5% bovine serum albumin (BSA) for 1 h at room temperature to reduce nonspecific binding. The samples were incubated with primary antibody overnight at 4 °C. On the following day, samples were equilibrated to room temperature for 30 min, and the primary antibody was removed. After washing three times with PBS, cells were incubated with Alexa Fluor 488-conjugated secondary antibody for 1 h at room temperature in the dark. After further PBS washes, nuclei were counterstained with DAPI, followed by mounting. Fluorescence images were captured using a confocal microscope to assess the intracellular localization of β-catenin.

### 2.14 Response status of HCC patients to TACE or sorafenib treatment

Cancer treatment by transcatheter arterial chemoembolization (TACE) and the tyrosine kinase inhibitor sorafenib can yield clinical benefits, yet only a subset of patients respond to therapy. In the GSE104580 dataset, 147 patients with hepatocellular carcinoma (HCC) received TACE, among whom 81 were responders and 66 were non-responders. Among 67 patients in the GSE109211 dataset treated with sorafenib, 21 were responders and 46 were non-responders (18). This study evaluated the differential response profiles to TACE or sorafenib across these cohorts, aiming to determine the association between TOP2A expression and therapeutic outcomes.

### 2.15 Tumor immune dysfunction and exclusion score

The Tumor Immune Dysfunction and Exclusion (TIDE) computational method was developed to predict response to immune checkpoint blockade (ICB) by modeling two primary mechanisms of tumor immune evasion: T cell dysfunction in tumors with high cytotoxic T lymphocyte (CTL) infiltration, and prevention of T cell infiltration in tumors with low CTL levels (37). Based on this framework, the TIDEpy Python package (https://github.com/jingxinfu/TIDEpy) was utilized in this study to analyzes pre-treatment gene expression data and compute integrated TIDE scores, immune dysfunction scores, immune exclusion scores, and infiltration levels of immunosuppressive cells including cancer-associated fibroblasts (CAFs), myeloid-derived suppressor cells (MDSCs), and M2-type tumor-associated macrophages (TAMs.M2). Additionally, it provides other immune-relevant indicators such as PD-L1, IFNG, and CD8, which collectively reflect the activity of tumor-infiltrating immune cells. By integrating these diverse features, TIDE offers a comprehensive assessment of tumor immune evasion potential, where higher TIDE scores are associated with poorer responses to immune checkpoint inhibitor therapy. Accordingly, TIDE analysis was employed to evaluate differences in these immune-related indicators across hepatocellular carcinoma (HCC) subgroups and to predict patient-specific immunotherapy responsiveness.

### 2.16 Screening of TOP2A-sensitive drugs

This study utilized the pRRophetic R package (version 0.5) to preliminarily assess drug sensitivity in the TCGA-LIHC and ICGC-HCC datasets based on the known cell line expression matrix and drug sensitivity data from the CGP database. Given that this cell-line-based correlation analysis has inherent limitations in predicting clinical *in vivo* responses, it was employed here as a discovery-based screening tool rather than direct clinical evidence. Spearman correlation analysis was performed between TOP2A expression and the estimated drug sensitivity (IC_50_). Drugs significantly correlated with TOP2A were screened using the threshold of r < -0.5 and p < 0.05. The overlapping drugs identified in both the TCGA-LIHC and ICGC-HCC datasets were selected for further analysis. Molecular docking was performed using Autodock Vina to explore potential interactions between TOP2A proteins and drug molecules. These computational hypotheses provided a basis for selecting candidates to be validated in future biological experiments.

### 2.17 Drug sensitivity validation

To evaluate drug sensitivity, Huh7 and HepG2 cells were seeded in 96-well plates at a density of 1×10^4^ cells per well and allowed to adhere overnight. The cells were then treated with increasing concentrations of A-443654 (purchased from Selleck Chemicals) for 48 hours. The compound was initially dissolved in DMSO and subsequently diluted in culture medium to the desired working concentrations. Cell viability was quantified using the Cell Counting Kit-8 (CCK-8; Beyotime, China) according to the manufacturer’s protocol. Briefly, after the 48-hour treatment, 10 μL of CCK-8 reagent was added to each well, followed by incubation for 2 hours at 37 °C. The absorbance was measured at 450 nm, and the half-maximal inhibitory concentration (IC_50_) was calculated by nonlinear regression analysis using GraphPad Prism 9.0.

### 2.18 *In vivo* tumor xenograft experiments

Huh7 wild-type (WT) cells and TOP2A-knockdown Huh7 cells (5 × 10^6^ cells/mouse in 0.1 mL of a 50% Matrigel/50% saline solution) were subcutaneously injected into the inguinal region of 6-week-old female BALB/c nude mice (purchased from GemPharmatech, Jiangsu, China; n=5 per group). Mouse body weight and tumor size were measured every three days. Tumor burden was assessed 21 days post-inoculation. C57BL/6 mice were obtained from Jiangsu Jizhi Pharmaceutical (Jiangsu, China). For the subcutaneous xenograft model, 2 × 10^6^ Hepa 1–6 cells suspended in 100 μL of PBS were implanted into mice. When the average tumor volume reached approximately 100 mm³, the tumor-bearing mice were randomly divided into four groups (n=5 per group) and treated as follows: 1) Control; 2) TOP2A candidate inhibitor A-443654 (Selleck, 5 mg/kg, p.o., 5 times/week for 2 weeks); 3) Anti-PD-L1 monoclonal antibody Atezolizumab (Selleck, 5 mg/kg, i.p., twice weekly for 2 weeks); or 4) The combination of A-443654 and Atezolizumab. Body weight and tumor size were monitored every three days, and the tumor burden was evaluated at the endpoint (21 days after treatment initiation). All animal experiments were approved by the Institutional Animal Care and Use Committee of the First Hospital of Shanxi Medical University.

### 2.19 Statistics analysis

All data processing and analyses were performed using R software (version 4.4.0) and GraphPad Prism 9. Samples with incomplete clinical or survival data were excluded prior to downstream analyses. To ensure data quality, low-abundance genes (defined as those with expression counts > 1 in fewer than 10 samples) were filtered out. For all cohorts, patients were stratified into high- and low-expression groups based on the median expression of the target genes. For comparisons of two groups of continuous variables, independent Student’s t-tests were used for normally distributed variables, while the Mann-Whitney U test (Wilcoxon rank-sum test) was applied for non-normally distributed variables. Chi-square or Fisher’s exact tests were used to evaluate differences between categorical variables. The survival R package (version 3.5.8) was employed for survival analysis, and univariate Cox regression analysis was conducted to identify prognostic risk factors. All statistical P-values were two-sided, with P < 0.05 considered statistically significant.

## 3 Results

### 3.1 Differential expression, diagnostic value and prognostic significance of TOP2A in HCC

**Figure 2:**
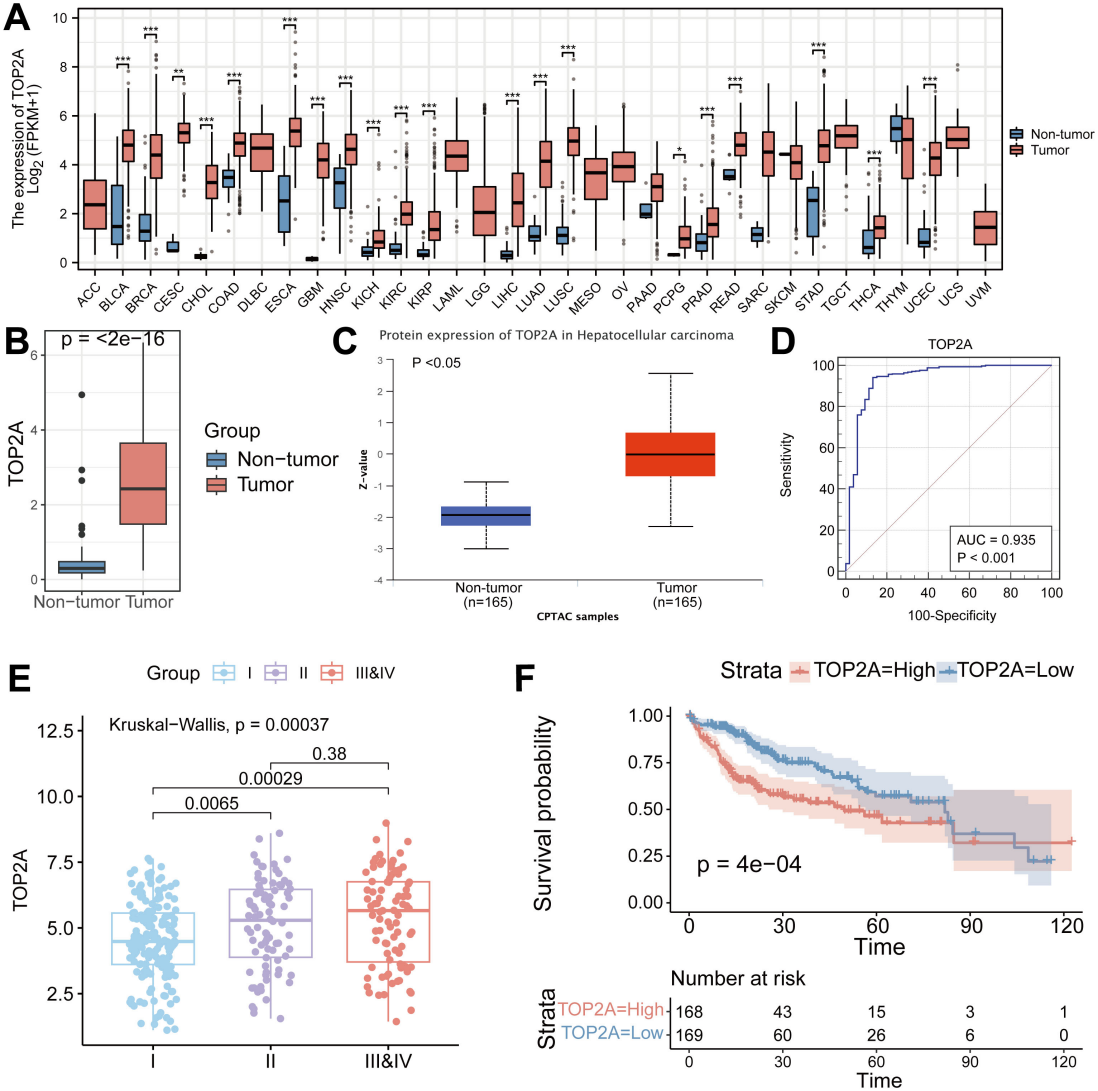
TOP2A is upregulated in HCC and associated with advanced disease and poor prognosis. **(A)** Pan-cancer analysis of TOP2A mRNA expression in tumor and normal tissues from the TCGA database. **(B)** TOP2A mRNA is significantly elevated in HCC tumor tissues compared with non-tumor controls in the TCGA-LIHC cohort (p < 0.05). **(C)** Consistent upregulation of TOP2A protein expression in HCC relative to adjacent normal tissues from the UALCAN database (p < 0.05). **(D)** ROC curve of the TCGA-LIHC cohort demonstrating the diagnostic utility of TOP2A expression for discriminating HCC from non-tumor tissues (AUC = 0.935, p < 0.05). **(E)** TOP2A expression increases with advancing clinical stage (I–IV) in HCC patients from the TCGA-LIHC dataset (p < 0.05, Kruskal–Wallis test). **(F)** Kaplan–Meier analysis based on TCGA-LIHC data reveals that high TOP2A expression correlates with significantly shorter overall survival (p < 0.05). Data are presented as mean ± SEM; *p < 0.05, **p < 0.01, ***p < 0.001

Analysis across 33 cancer types from the TCGA database revealed that TOP2A expression was significantly elevated in the majority of tumors compared to non-tumor tissues (**Figure 2A**), with a notable increase observed in HCC (**Figure 2B**). Consistent with this, protein-level analysis via the UALCAN database confirmed markedly higher TOP2A expression in HCC tissues than in non-HCC samples (**Figure 2C**). Receiver operating characteristic (ROC) analysis indicated strong diagnostic utility of TOP2A for HCC (AUC = 0.935, p < 0.05; **Figure 2D**). Furthermore, TOP2A expression increased significantly with advancing tumor stage (p < 0.05; **Figure 2E**). Elevated TOP2A expression was also associated with shorter overall survival (OS, p < 0.05; **Figure 2F**). These findings were corroborated in the ICGC-HCC validation cohort (**Supplementary Figure 1**).

**Figure 3:**
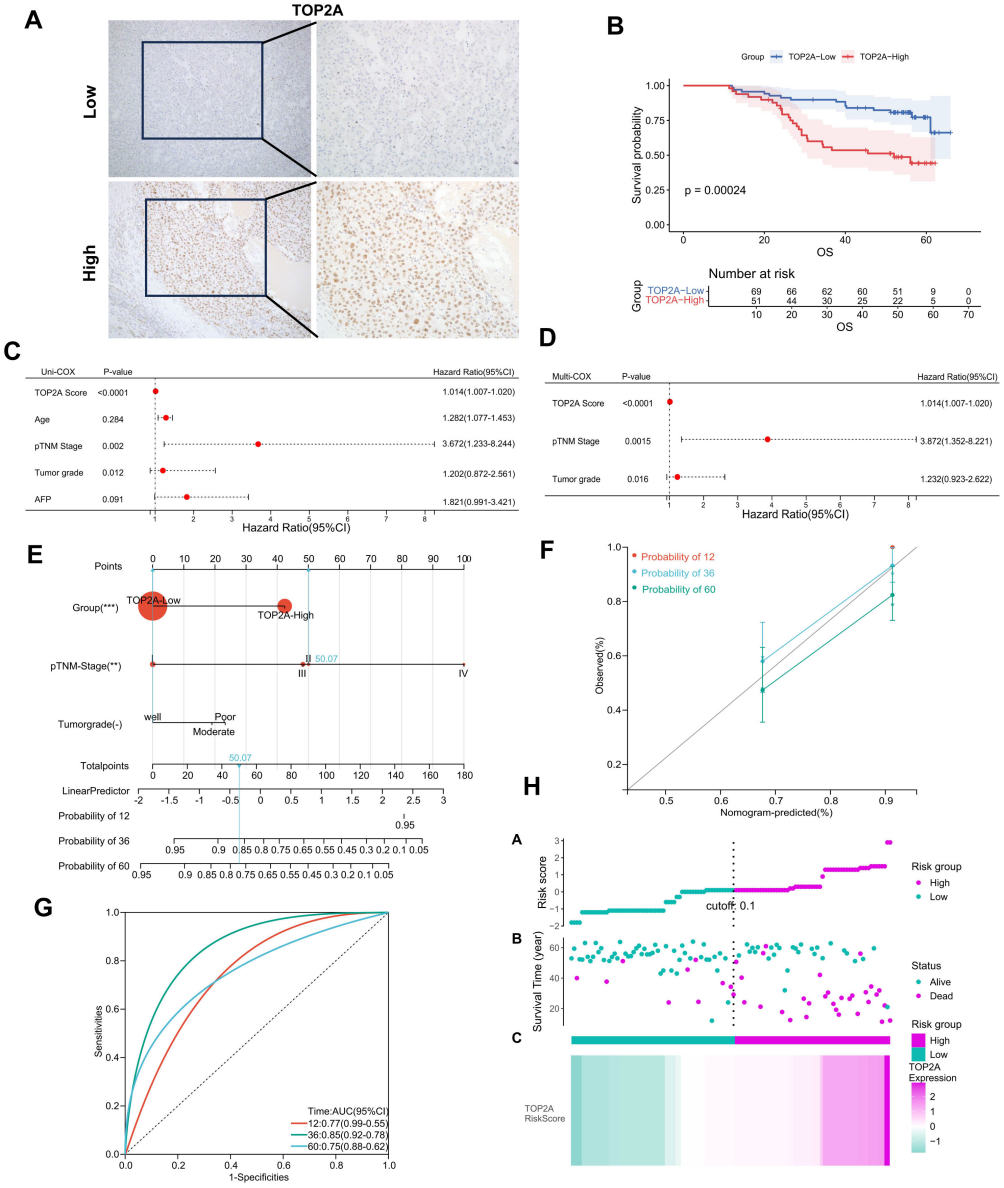
TOP2A serves as an independent prognostic biomarker in HCC. **(A)** Representative immunohistochemical (IHC) staining of TOP2A in HCC tissue sections. **(B)** Kaplan–Meier curve showing significantly worse overall survival in patients with high TOP2A expression (p < 0.05). **(C, D)** Univariate **(C)** and multivariate **(D)** Cox regression analyses identifying TOP2A as an independent prognostic factor for overall survival. **(E)** Nomogram integrating TOP2A IHC score, TNM stage, and tumor grade for predicting overall survival. **(F)** Calibration curve evaluating the consistency between predicted and observed survival outcomes. **(G)** ROC curve assessing the predictive accuracy of the prognostic model. **(H)** Distribution of risk scores, survival status, and TOP2A expression levels across the patient cohort.

To validate the TOP2A expression patterns observed in the TCGA and ICGC databases, we performed immunohistochemistry (IHC) on a cohort of HCC tissues. Representative IHC images demonstrating varying staining intensities of TOP2A are presented in **Figure 3A**, and the corresponding clinical baseline characteristics of the cohort are summarized in **Table 1**. Survival analysis revealed that patients with high TOP2A expression (n = 51) had significantly shorter OS compared to those with low expression (p < 0.05; **Figure 3B**). Univariate and multivariate Cox regression analyses identified TOP2A as an independent prognostic factor for OS in HCC (**Figures 3C, D**). After adjustment for age, tumor grade, pTNM stage, and alpha-fetoprotein (AFP) levels, the TOP2A IHC score remained significantly associated with survival (HR = 1.014, 95% CI: 1.007–1.020, p < 0.05). Using these independent prognostic indicators, we developed a nomogram to predict 1−, 3−, and 5−year OS probabilities (**Figure 3E**). The model exhibited good discriminative ability, with a C-index of 0.76 (95% CI: 0.69–0.84, p = 6.6 × 10^-^¹³). Calibration curves showed high consistency between predicted and observed outcomes for 3− and 5−year OS, while the 1−year OS prediction demonstrated some deviation, possibly due to the limited sample size in this subgroup (**Figure 3F**). Time-dependent ROC analysis further validated the model’s predictive performance, with AUC values of 0.77 (95% CI: 0.55–0.99), 0.85 (95% CI: 0.78–0.92), and 0.75 (95% CI: 0.62–0.88) for 1−, 3−, and 5−year OS, respectively (**Figure 3G**). Finally, the distribution of the model risk scores, patient survival status, and TOP2A expression levels are depicted in **Figure 3H**. The analysis showed that higher risk scores were associated with increased mortality and reduced survival time, reinforcing the prognostic value of TOP2A expression in HCC.

**Table 1:** Clinical characteristics of 120 patients with HCC.

Characteristics	Total	TOP2A-low	TOP2A-high	P
	N=120 (%)	N=69 (%)	N=51 (%)	
Age				0.62
Mean ± SD	57.17 ± 7.58	58.09 ± 8.14	55.94 ± 6.62	
Median (min-max)	58 (35,72)	59 (35,72)	55 (38,68)	
Gender				0.53
Female	64(53.33%)	39(56.52%)	25(49.02%)	
Male	56(46.67%)	30(43.48%)	26(50.98%)	
ECOG				0.42
0	49(40.83%)	30 (43.48%)	19 (37.25%)	
1	71(59.17%)	39 (56.52%)	32 (62.75%)	
pTNM-Stage				0.19
I	59(49.17%)	35 (50.72%)	24 (47.06%)	
II	14(11.67%)	7 (10.14%)	7 (13.73%)	
III	44(36.67%)	27 (39.13%)	17 (33.33%)	
IV	3(2.50%)	0 (0.00%)	3 (5.88%)	
Tumor Grade				0.84
Well	42(35.00%)	26 (37.68%)	16(31.37%)	
Moderate	58(48.33%)	32 (46.38%)	26 (50.98%)	
Poor	20(16.67%)	11 (15.94%)	9 (17.65%)	
AFP (ng/ml)				0.58
<7	73 (60.83%)	40 (57.98%)	33 (64.71%)	
>7	47 (39.17%)	29 (42.02%)	18 (35.29%)	
OS				0.0017
Live	79 (65.83%)	54 (78.26%)	25 (49.02%)	
Dead	41 (34.17%)	15 (21.74%)	26 (50.98%)	

### 3.2 TOP2A promotes malignant progression of HCC

**Figure 4:**
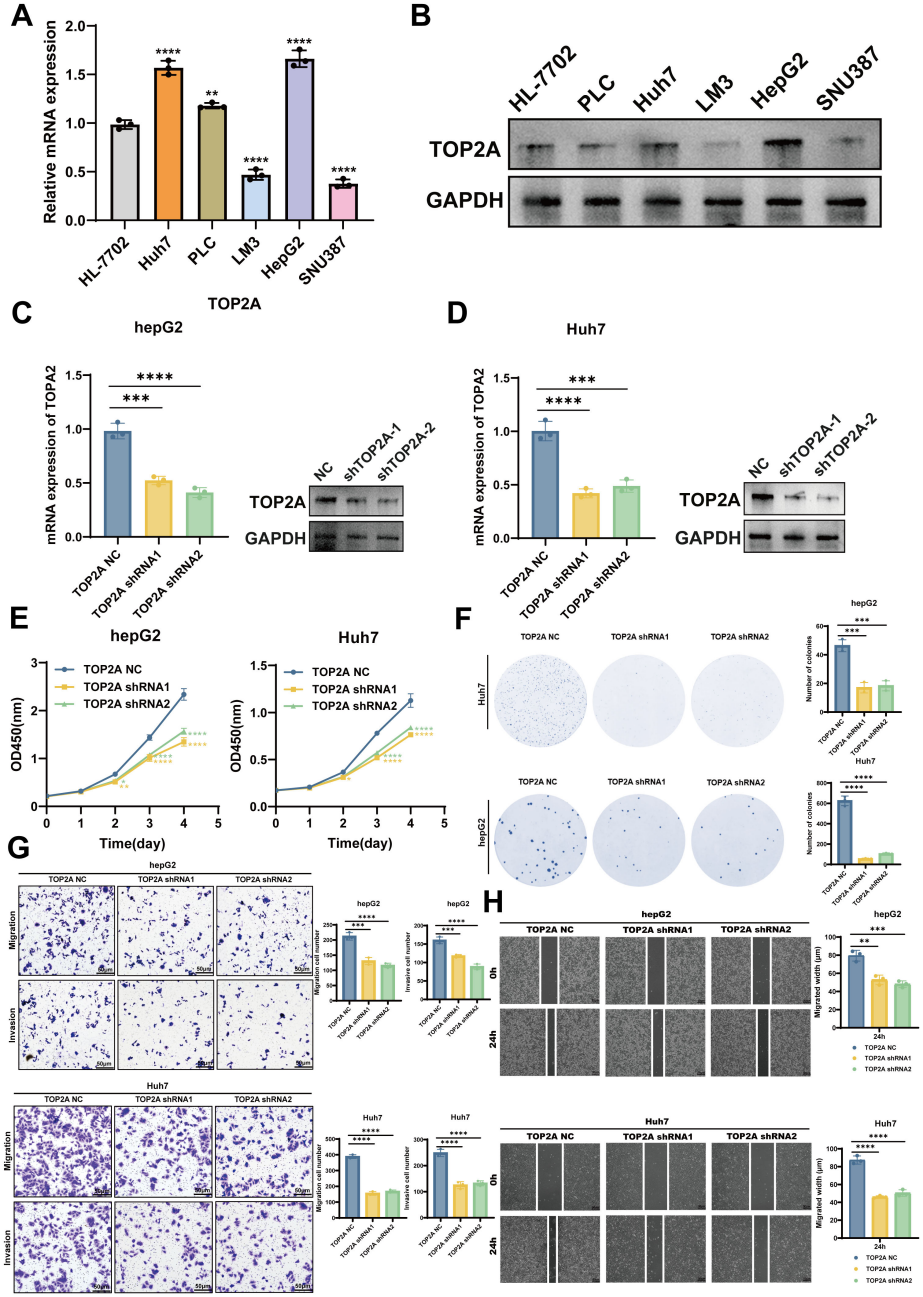
Knockdown of TOP2A suppresses proliferation, migration, and invasion in hepatocellular carcinoma cells. **(A)** TOP2A mRNA expression levels in different HCC cell lines measured by RT−qPCR. **(B)** TOP2A protein expression in HCC cell lines detected by Western blot. **(C, D)** TOP2A mRNA and protein expression in **(C)** and **(D)** cells after transduction with TOP2A−targeting shRNA (shRNA1 or shRNA2) or empty vector (PCDH). **(E)** Cell viability assessed by CCK−8 assay 48 hours after transfection. **(F)** Colony formation ability of cells transfected with TOP2A shRNA or control after two weeks of culture. **(G)** Cell invasion and migration evaluated by Transwell assay 48 hours post−transfection. **(H)** Cell migration measured by wound healing assay; images were captured at 0 and 24 hours. Data are presented as mean ± SEM; *p < 0.05, **p < 0.01, ***p < 0.001, ****p < 0.0001.

To elucidate the functional significance of TOP2A in HCC, we began by examining its expression in HCC cell lines. RT-qPCR and Western blot analyses consistently demonstrated that TOP2A was significantly upregulated at both the mRNA and protein levels in the majority of HCC cell lines relative to normal hepatic cells (**Figures 4A, B**). To explore its functional role, we generated stable TOP2A-knockdown models in HepG2 and Huh7 cells via lentivirus-mediated RNA interference. Knockdown efficiency was validated at both transcriptional and translational levels (**Figures 4C, D**). Subsequent functional experiments revealed that TOP2A depletion markedly suppressed malignant behaviors. Specifically, CCK-8 and colony formation assays indicated a significant decrease in cellular proliferation and clonogenic survival (**Figures 4E, F**). Moreover, Transwell and wound healing assays showed that TOP2A knockdown strongly inhibited the migratory and invasive capacities of HCC cells (**Figures 4G, H**). Considering the hepatoblastoma-like characteristics of HepG2 cells, we additionally performed TOP2A knockdown experiments in the highly metastatic HCCLM3 cell line to validate the generalizability of its pro-tumorigenic effects in HCC cells. Consistent with the observations in HepG2 and Huh7 cells, TOP2A silencing significantly suppressed the proliferative, migratory, and invasive capacities of HCCLM3 cells (**Supplementary Figure 2**). Together, these results establish TOP2A as a critical promoter of HCC aggressiveness, influencing proliferation, clonogenicity, migration, and invasion.

### 3.3 Diagnostic and prognostic significance of E2F6 in HCC

**Figure 5:**
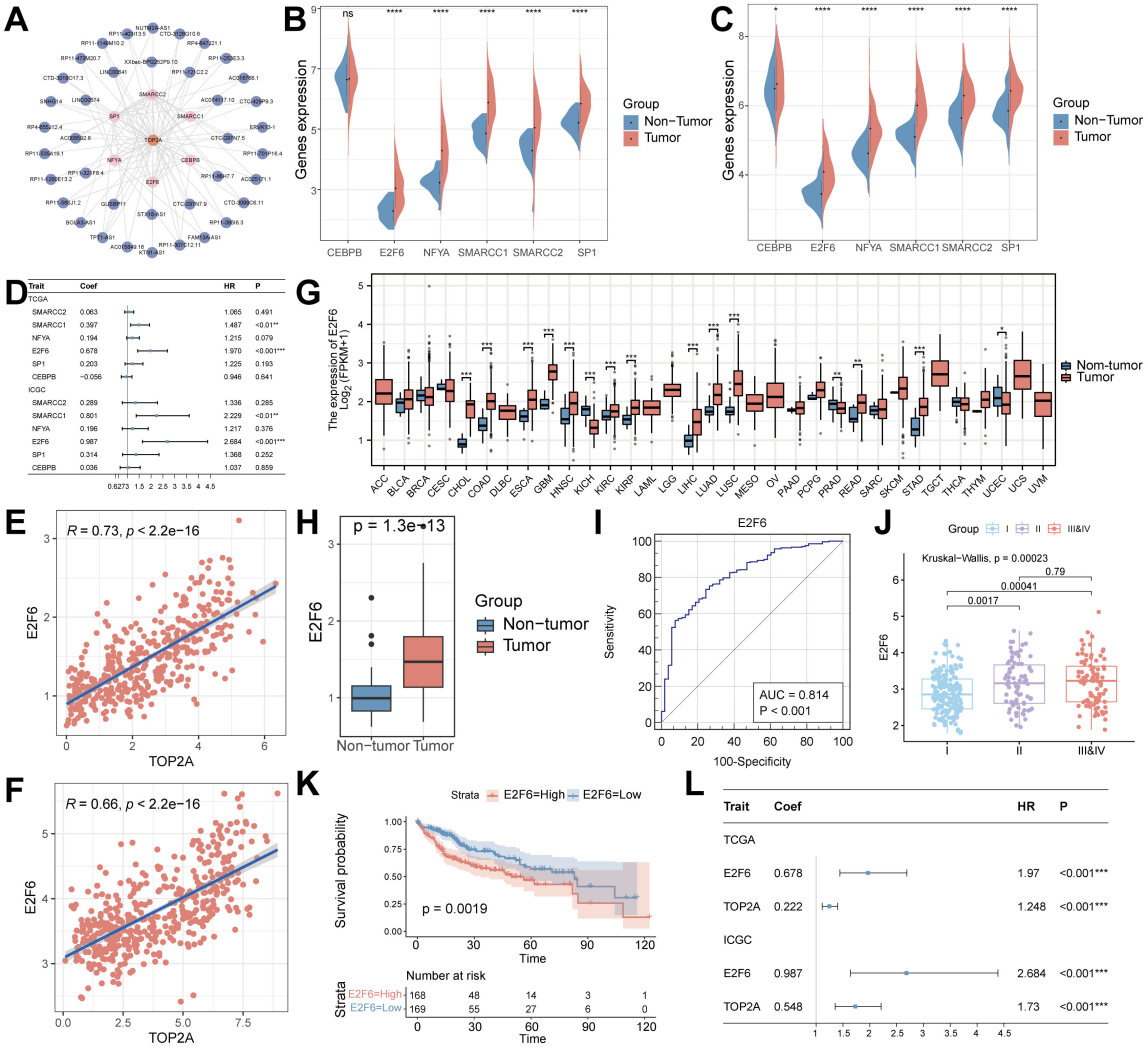
TOP2A−associated transcriptional network, the diagnostic and prognostic significance of E2F6 in HCC. **(A)** Regulatory network of TOP2A−related transcription factors (pink nodes) and lncRNAs (blue nodes) generated from the LncMAP database. **(B, C)** Violin plots displaying the expression of six transcription factors (SMARCC2, SMARCC1, NFYA, E2F6, SP1, and CEBPB) between Tumor and non−Tumor tissues in the TCGA−LIHC **(B)** and ICGC−HCC **(C)** cohorts. **(D)** Forest plot of univariate Cox regression analysis evaluating the association between the six transcription factors and overall survival in HCC. **(E, F)** Positive correlation between E2F6 and TOP2A expression in the TCGA−LIHC **(E)** and ICGC−HCC **(F)** cohorts (Pearson correlation). **(G)** Pan−cancer expression profile of E2F6 mRNA from TCGA. **(H)** E2F6 is significantly overexpressed in HCC compared with non−tumor tissues (p < 0.05, Wilcoxon test). **(I)** ROC analysis of E2F6 for discriminating HCC from normal liver tissues (AUC = 0.814, p < 0.05). **(J)** E2F6 expression across HCC clinical stages (p < 0.05, Kruskal–Wallis test). **(K)** Kaplan–Meier curves showing poorer overall survival in patients with high E2F6 expression (p < 0.05). **(L)** Forest plot of univariate Cox regression analysis for E2F6 and TOP2A in the TCGA-LIHC and ICGC-HCC cohorts. (p < 0.05). Data are presented as mean ± SEM; ***p < 0.001, ****p < 0.0001.

Based on the LncMAP web tool analysis, six transcription factors (TFs)—SMARCC2, SMARCC1, NFYA, E2F6, SP1, and CEBPB—were identified as potential regulators of TOP2A (**Figure 5A**). Differential expression analysis revealed that five of these TFs (SMARCC2, SMARCC1, NFYA, E2F6, and SP1) were significantly dysregulated in HCC tissues compared with non-tumor tissues across both the TCGA-LIHC and ICGC-HCC cohorts (**Figures 5B, C**). Univariate Cox regression analysis further indicated that only SMARCC1 and E2F6 were significantly associated with HCC prognosis (**Figure 5D**). Between these two candidates, only E2F6 had well-documented transcription factor binding sites annotated in the JASPAR database. Therefore, E2F6 was selected for further investigation to explore its transcriptional regulatory relationship with TOP2A. Correlation analyses using data from both the TCGA-LIHC and ICGC-HCC consistently revealed a strong positive relationship between E2F6 and TOP2A expression (TCGA-LIHC: R = 0.73, p < 0.05, **Figure 5E**; ICGC-HCC: R = 0.66, p < 0.05, **Figure 5F**), supporting its potential role as a transcription factor of TOP2A.

Having established a strong positive correlation between E2F6 and TOP2A, we next explored the clinical relevance of E2F6 itself in HCC. Analysis of the TCGA database revealed significantly elevated expression of E2F6 in tumor tissues compared to non-tumor tissues across multiple cancer types (**Figure 5G**), including HCC (**Figure 5H**). ROC analysis based on TCGA-LIHC data demonstrated that E2F6 possesses high diagnostic value for HCC (AUC = 0.814, p < 0.05; **Figure 5I**). Mirroring the patterns observed for TOP2A, E2F6 expression levels increased with advancing tumor stages (p < 0.05, **Figure 5J**), and higher E2F6 expression was significantly associated with shorter OS (**Figure 5K**). The diagnostic and prognostic capacity of E2F6 was further validated in the independent ICGC-HCC cohort (**Supplementary Figure 3**). Furthermore, univariate Cox regression analysis confirmed both E2F6 and TOP2A as significant risk factors for OS in HCC patients (**Figure 5L**), reinforcing their prognostic relevance in HCC.

### 3.4 E2F6 directly binds to TOP2A promoter and activates its transcription

**Figure 6:**
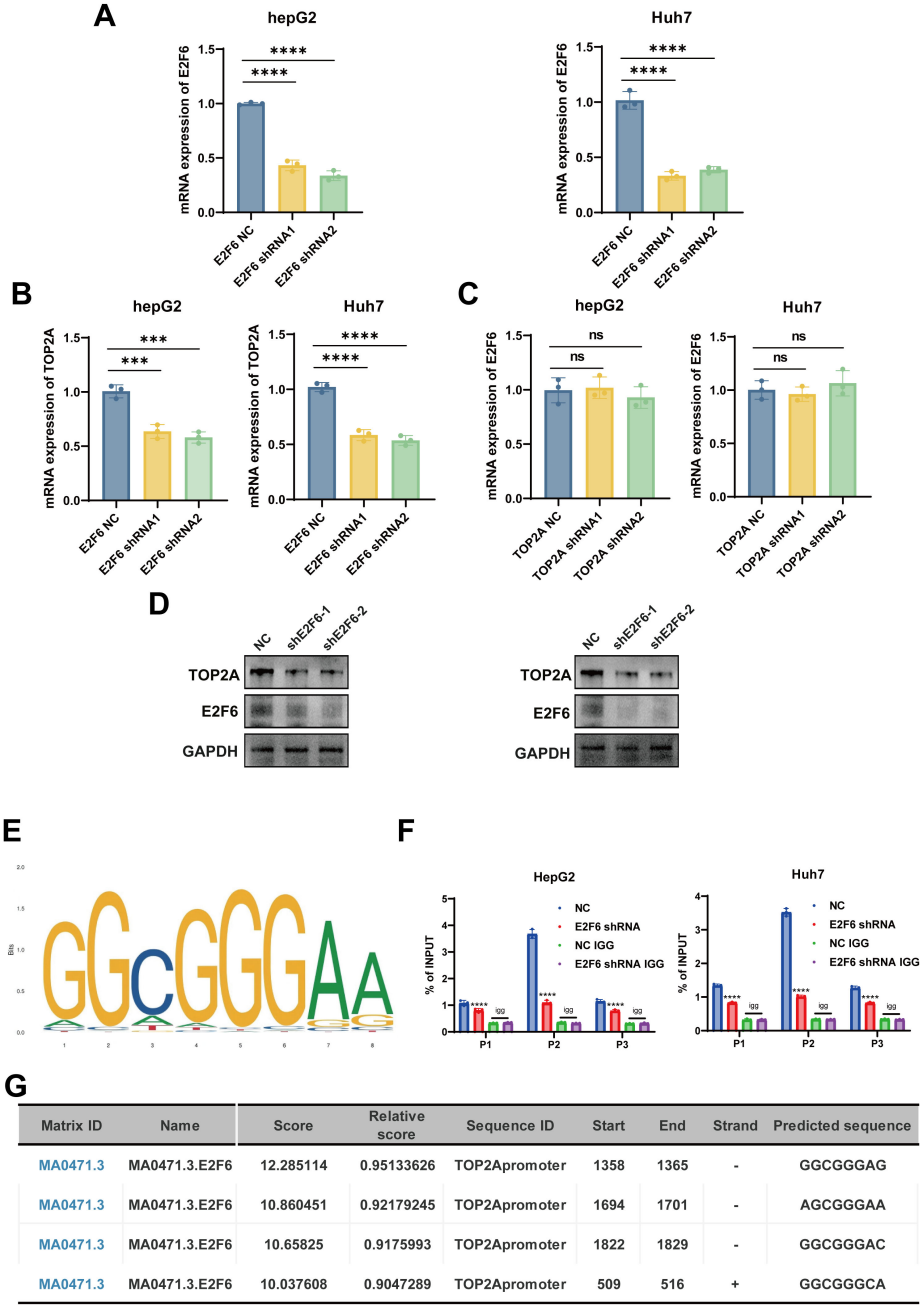
E2F6 transcriptionally regulates TOP2A by directly binding to its promoter. **(A)** Knockdown efficiency of E2F6 in HepG2 and Huh7 cells measured by RT−qPCR. **(B)** TOP2A mRNA expression following E2F6 knockdown in HepG2 and Huh7 cells. **(C)** E2F6 mRNA expression after TOP2A knockdown in HepG2 and Huh7 cells. **(D)** TOP2A protein levels upon E2F6 knockdown in HepG2 and Huh7 cells analyzed by Western blot. **(E)** Predicted binding motif of E2F6 from the JASPAR database. **(F)** ChIP−qPCR showing E2F6 enrichment at the TOP2A promoter region in control versus E2F6 knockdown cells. **(G)** JASPAR-predicted binding sites of E2F6 on the TOP2A promoter. Data are presented as mean ± SEM; ***p < 0.001, ****p < 0.0001. RT-qPCR, real-time quantitative PCR.

Based on our previous analysis, we further verified the transcriptional regulatory relationship between E2F6 and TOP2A. Initially, lentivirus-mediated stable knockdown of E2F6 was established in HepG2 and Huh7 cells, with the efficiency of knockdown verified at the mRNA level (**Figure 6A**). Subsequent qRT-PCR analysis showed that E2F6 silencing significantly reduced TOP2A mRNA expression (**Figure 6B**). In contrast, knockdown of TOP2A did not significantly affect E2F6 mRNA levels (**Figure 6C**), supporting a unidirectional regulatory relationship. Consistent with this, western blot results further confirmed that E2F6 knockdown led to decreased TOP2A protein expression (**Figure 6D**). To further elucidate the transcriptional mechanism, potential E2F6 binding motifs were predicted within the TOP2A promoter by the JASPAR database (**Figures 6E, G**). Among the predicted binding sites, the site with the highest predicted score should be given priority for examination. Subsequent chromatin immunoprecipitation quantitative PCR (ChIP-qPCR) detection confirmed that E2F6 directly bound to the promoter region of TOP2A (**Figure 6F**). In conclusion, these results indicate that E2F6 plays a key role in the transcriptional regulation of TOP2A.

### 3.5 Identification and validation of a TOP2A/E2F6 co-high expression subtype in HCC with functional enrichment analysis

**Figure 7:**
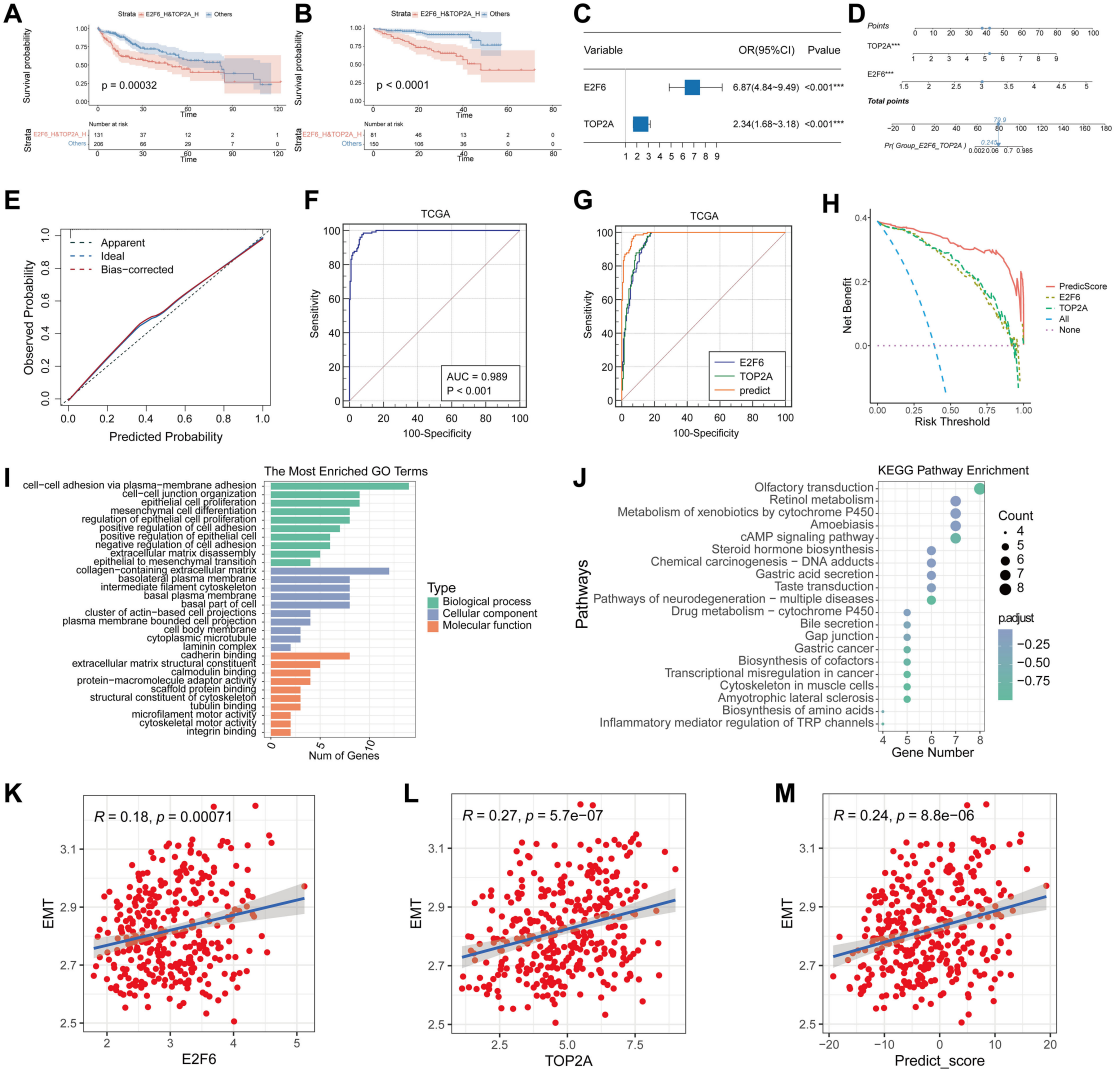
Prognostic implication, predictive modeling, and functional enrichment of concurrent high expression of E2F6 and TOP2A in hepatocellular carcinoma. **(A)** Kaplan–Meier survival analysis of HCC patients from the TCGA−LIHC cohort, stratified by concurrent high expression of E2F6 and TOP2A versus others, showing significantly poorer prognosis in the dual-high expression group (p < 0.05). **(B)** Validation in the ICGC−HCC cohort: Kaplan–Meier analysis confirms that patients with concurrent high expression of E2F6 and TOP2A exhibit significantly worse survival outcomes (p < 0.05). **(C)** Multivariable logistic regression model based on TCGA−LIHC data for predicting patients with dual high−expression of E2F6 and TOP2A, visualized using a forest plot. (E2F6: OR = 6.87, 95% CI = 4.84–9.49; TOP2A: OR = 2.34, 95% CI = 1.68–3.18, p < 0.05). **(D)** Nomogram integrating E2F6 and TOP2A expression for individualized prediction of dual high-expression probability (p < 0.05). **(E)** Calibration curve demonstrating good agreement between predicted probabilities and actual observations of the dual high-expression phenotype. **(F)** Receiver operating characteristic (ROC) curve evaluation of the combined model, showing excellent discriminative ability (AUC = 0.989, p < 0.05) in identifying patients with concurrent high expression of E2F6 and TOP2A. **(G)** Comparative ROC analysis of the combined model versus E2F6 or TOP2A alone, demonstrating enhanced predictive performance of the combined approach. **(H)** Decision curve analysis (DCA) showing superior clinical utility and net benefit of the combined model across various risk thresholds compared to single-gene models. **(I)** Gene Ontology (GO) enrichment analysis of differentially expressed genes between the E2F6/TOP2A dual high−expression group and other patients in the TCGA−LIHC cohort. **(J)** KEGG pathway enrichment analysis revealing significantly enriched pathways associated with the dual high-expression phenotype. **(K)** Scatter plot revealing a significant positive correlation between E2F6 expression and EMT scores (R = 0.18, p < 0.05), further supporting the involvement of E2F6 in the EMT process within the TCGA-LIHC cohort. **(L)** Scatter plot demonstrating a significant positive correlation between TOP2A expression levels and EMT scores in the TCGA-LIHC cohort (R = 0.27, p < 0.05), indicating an association between elevated TOP2A expression and enhanced epithelial-mesenchymal transition. **(M)** Scatter plot illustrating a significant positive correlation between the predictive risk score and EMT scores (R = 0.24, p < 0.05), reinforcing the link between the model’s risk stratification capacity and EMT status in the TCGA-LIHC cohort.

Based on E2F6 and TOP2A expression levels, HCC patients were categorized into two groups: those with high expression of both genes (E2F6_H&TOP2A_H) and all others. Patients in the E2F6_H&TOP2A_H group showed significantly shorter OS and poorer prognosis compared to other HCC patients (p < 0.05; **Figures 7A, B**). Given the unfavorable prognosis associated with this subgroup, accurate identification of these patients is crucial for implementing targeted therapeutic strategies to improve clinical outcomes. To facilitate this, a predictive model was developed using multivariate logistic regression based on E2F6 and TOP2A expression levels: PredictScore = 6.8719 × E2F6 + 2.3388 × TOP2A (**Figure 7C**). A nomogram was constructed to visualize the model (**Figure 7D**), and its predictive performance was evaluated using calibration curves, which demonstrated strong agreement between predicted and actual outcomes (**Figure 7E**). ROC analysis further confirmed the model’s excellent discriminative ability, with AUC values of 0.989 in the TCGA-LIHC cohort (**Figure 7F**). Moreover, the model outperformed alternative approaches in identifying E2F6_H&TOP2A_H patients (**Figure 7G**). Decision curve analysis (DCA) also supported its clinical applicability (**Figure 7H**).

To validate the predictive accuracy of the established model, we performed external validation using three independent cohorts: ICGC-HCC, GSE109211, and GSE104580. The model consistently demonstrated strong performance in identifying E2F6_H&TOP2A_H patients, with AUC values of 0.985 (ICGC-HCC, **Supplementary Figure 4A**), 0.810 (GSE109211, **Supplementary Figure 4B**), and 0.937 (GSE104580, **Supplementary Figure 4C**). DCA further confirmed the model’s clinical utility, showing superior net benefit across all validation cohorts (**Supplementary Figures 4D–F**). In summary, the model exhibits robust and consistent performance in accurately discriminating E2F6_H&TOP2A_H patients across TCGA, ICGC, and GEO datasets, underscoring its generalizability and potential for clinical translation.

To further investigate the functional implications of concurrent high expression of E2F6 and TOP2A in hepatocellular carcinoma (HCC) progression, we performed differential gene expression analysis using the TCGA-LIHC dataset, comparing patients with high expression of both E2F6 and TOP2A against other HCC patients. This analysis identified 597 differentially expressed genes (|logFC| > 2, adj. p<0.05, **Supplementary Table 4**), which were subsequently subjected to GO and KEGG enrichment analyses. GO enrichment analysis revealed that these differentially expressed genes were significantly enriched in several biological processes (BP), primarily associated with cell-cell adhesion via plasma-membrane adhesion molecules, cell-cell junction organization, epithelial cell proliferation, and mesenchymal cell differentiation. For cellular components (CC), the most significantly enriched terms included collagen-containing extracellular matrix, basolateral plasma membrane, intermediate filament cytoskeleton, and basal plasma membrane. Molecular functions (MF) that showed significant enrichment comprised cadherin binding, extracellular matrix structural constituent, and calmodulin binding. These enriched terms collectively indicate substantial alterations in extracellular matrix composition, cell adhesion mechanisms, and proliferative processes, strongly suggesting that high expression of E2F6 and TOP2A may promote HCC progression through enhancing epithelial-mesenchymal transition (EMT), proliferation, and metastasis (**Figure 7I**, **Supplementary Table 5**). KEGG pathway analysis further demonstrated that the 597 differentially expressed genes were significantly associated with multiple cancer-related pathways, including retinol metabolism, metabolism of xenobiotics by cytochrome P450, cAMP signaling pathway, steroid hormone biosynthesis, and chemical carcinogenesis-DNA adducts (**Figure 7J**; **Supplementary Table 6**).

Furthermore, we explored the relationship between E2F6/TOP2A expression and epithelial-mesenchymal transition (EMT). The results demonstrated that both E2F6 and TOP2A expression levels, as well as the combined predictive score derived from these two markers, were significantly positively correlated with EMT activation (**Figures 7K–M**). These findings further support the conclusion that E2F6 promotes EMT in HCC through transcriptional regulation of TOP2A.

### 3.6 DKK1 was identified as a key gene mediating TOP2A-regulated EMT

**Figure 8:**
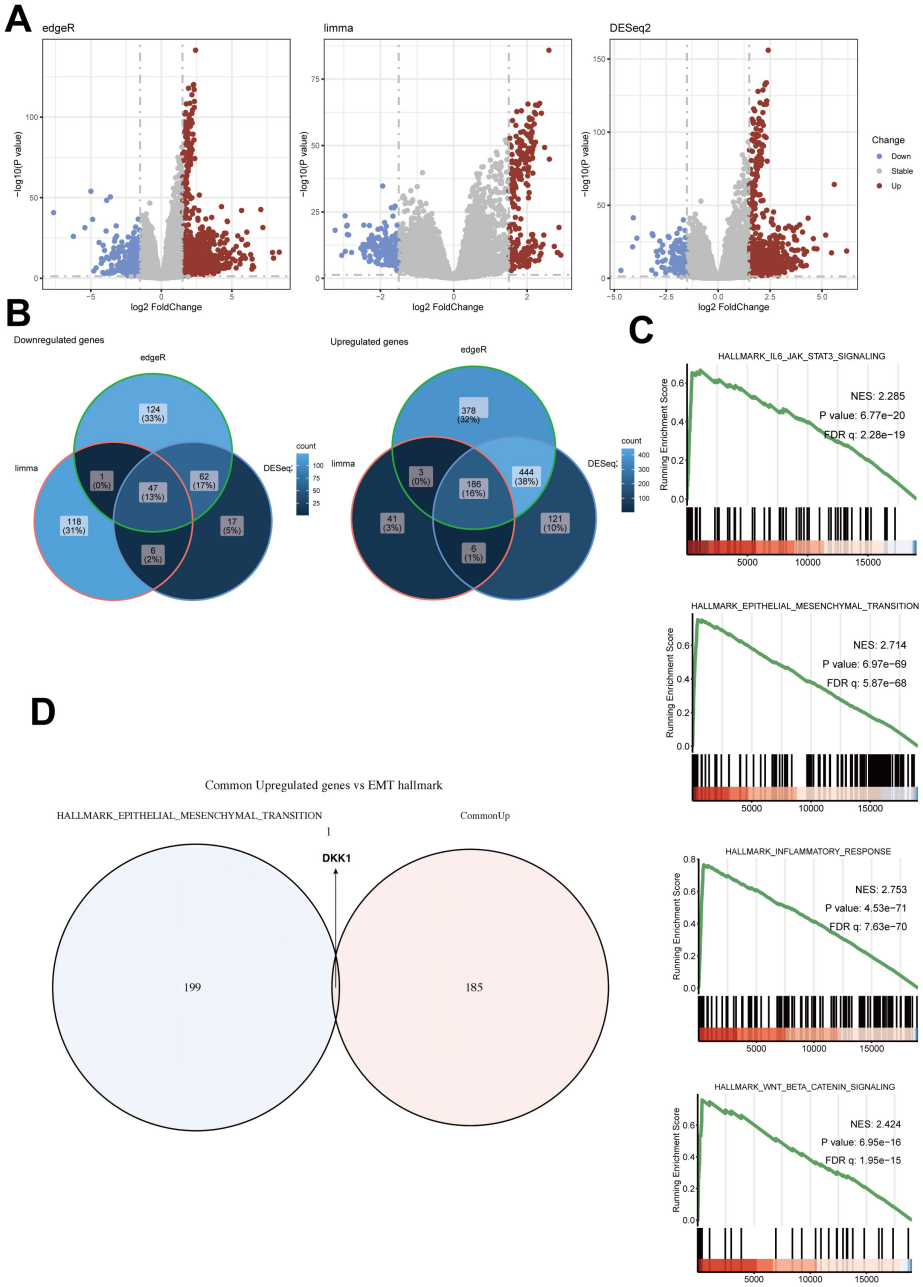
Identification of TOP2A downstream genes in the TCGA-LIHC cohort. **(A)** Volcano plots depicting differentially expressed genes (DEGs) between TOP2A-high and TOP2A-low groups using three independent methods: edgeR (left), limma (middle), and DESeq2 (right). Red and blue dots represent up- and down-regulated genes, respectively; gray dots denote genes with no significant change. **(B)** Venn diagrams showing the overlap of downregulated (left) and upregulated (right) DEGs identified by edgeR, limma, and DESeq2. The intersecting regions indicate the proportion of consistently dysregulated genes across all three methods. **(C)** Gene Set Enrichment Analysis (GSEA) of Hallmark gene sets performed using the gene list ranked by log_2_(fold change), with normalized enrichment score (NES), nominal p-value, and false discovery rate (FDR) indicated for significantly enriched terms. **(D)** Overlap between the commonly upregulated DEGs and the “Epithelial-Mesenchymal Transition” (EMT) Hallmark gene set. The candidate downstream gene DKK1 is highlighted within the shared gene set.

To elucidate the key downstream mechanisms by which TOP2A promotes malignant progression through regulating EMT in HCC, we conducted a comprehensive differential expression analysis. Samples were stratified into high and low TOP2A expression groups and analyzed using three widely used bioinformatics algorithms: edgeR, limma, and DESeq2 (**Figure 8A**). Integration of the results identified 47 consistently downregulated and 186 consistently upregulated genes (|logFC| > 1.5, adj. p<0.05, **Figure 8B**). Subsequent Gene Set Enrichment Analysis (GSEA) revealed significant enrichment of gene sets associated with inflammatory response, IL-6–JAK–STAT3 signaling, EMT, and WNT–β-catenin signaling pathways (**Figure 8C**). These findings align with previous studies and further confirm TOP2A’s role in promoting HCC progression via EMT processes. By intersecting the TOP2A-associated upregulated genes with a well-established EMT signature gene set, we identified DKK1 as a critical downstream gene through which TOP2A regulates EMT (**Figure 8D**).

### 3.7 TOP2A drives HCC progression via DKK1

**Figure 9:**
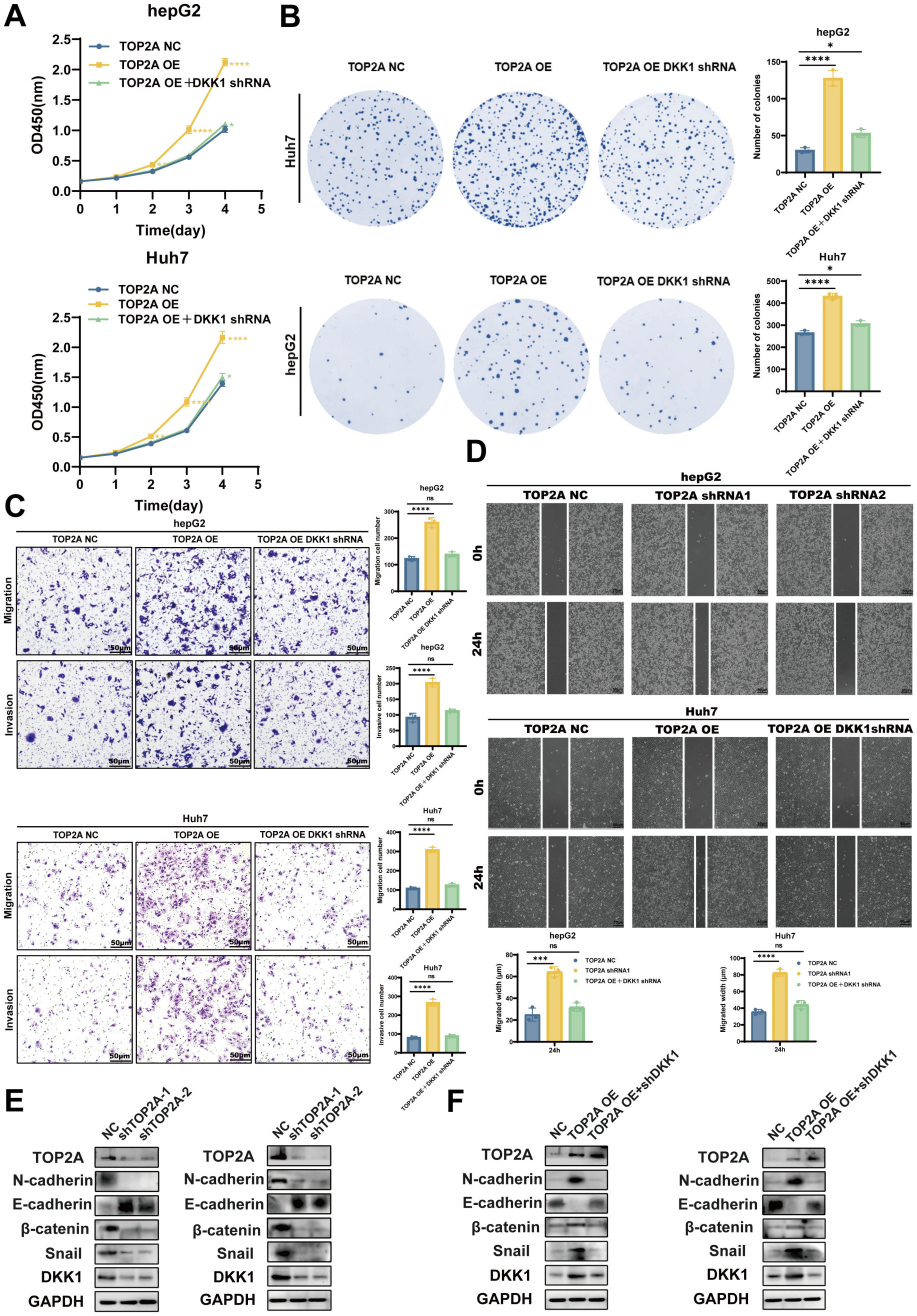
TOP2A promotes malignant progression of HCC by regulating DKK1. **(A)** Cell viability of HepG2 and Huh7 cells was assessed by CCK-8 assay 48 hours after transfection with TOP2A PCDH, TOP2A OE, or TOP2A OE combined with DKK1 shRNA. **(B)** Colony formation assays were performed to evaluate the proliferative ability of transfected HepG2 and Huh7 cells (TOP2A PCDH, TOP2A OE, or TOP2A OE + DKK1 shRNA) following 2 weeks of culture in complete DMEM medium. **(C)** Transwell assays were used to examine the invasion and migration capacities of HepG2 and Huh7 cells 48 hours after transfection with TOP2A PCDH, TOP2A OE, or TOP2A OE combined with DKK1 shRNA. **(D)** Wound healing assays were conducted to assess the migration of HepG2 and Huh7 cells transfected with TOP2A PCDH, TOP2A OE, or TOP2A OE together with DKK1 shRNA. Images were taken at 0 and 24 hours. **(E)** Western blot analysis of EMT-related markers (β-catenin, E-cadherin, N-cadherin, Snail) in HepG2 and Huh7 cells transfected with TOP2A shRNA. **(F)** Western blot analysis showing changes in EMT marker expression in HepG2 and Huh7 cells co-transfected with TOP2A OE and DKK1 shRNA. Data are presented as mean ± SEM; *p < 0.05, **p < 0.01, ***p < 0.001, ****p < 0.0001.

To further elucidate the functional role of DKK1 in TOP2A-mediated malignant progression, we next performed *in vitro* functional assays using control cells, TOP2A-overexpressing cells, and TOP2A-overexpressing cells with DKK1 knockdown. The overexpression and knockdown efficiencies had been confirmed previously in both Huh7 and HepG2 cell lines (**Supplementary Figure 5**). CCK-8 and colony formation assays demonstrated that DKK1 knockdown significantly attenuated the enhanced proliferative and clonogenic capacities induced by TOP2A overexpression, restoring these capabilities to near baseline levels (**Figures 9A, B**). Moreover, Transwell and wound healing assays showed that the promotive effects of TOP2A overexpression on cell migration and invasion were markedly suppressed upon DKK1 knockdown, reducing cell motility to a level comparable with the control group (**Figures 9C, D**). These results suggest that DKK1 is essential for TOP2A-driven proliferative and metastatic phenotypes in HCC. To further strengthen the robustness of these findings and considering the reported limitations of HepG2 as a representative HCC model, key functional assays were additionally validated in the HCCLM3 cell line. The results were highly consistent with those observed in Huh7 and HepG2 cells, further supporting the role of the TOP2A/DKK1 axis in regulating HCC malignant phenotypes (**Supplementary Figure 6**).

Building on the functional evidence that DKK1 mediates TOP2A-driven phenotypes in hepatocellular carcinoma (HCC), we next sought to delineate the underlying molecular mechanism. Previous studies have reported that in HCC, DKK1 upregulates β-catenin expression to promote malignant progression (38). To further validate the status of β-catenin, immunofluorescence analysis was performed to examine its subcellular localization. The results showed that DKK1 knockdown decreased cytoplasmic β-catenin levels and reduced its nuclear accumulation, whereas DKK1 overexpression increased cytoplasmic β-catenin and promoted its nuclear translocation in both Huh7 and HepG2 cells (**Supplementary Figure 7**). To examine whether this axis is functionally linked to TOP2A, we detected and measured changes in key EMT-related markers and β-catenin by Western blot. Our results demonstrated that knockdown of TOP2A markedly reversed the expression of EMT-related proteins—such as E-cadherin, N-cadherin, and Snail—as well as β-catenin, indicating effective suppression of the EMT program (**Figure 9E**). Similarly, under TOP2A-overexpressing conditions, additional knockdown of DKK1 substantially inhibited the EMT phenotype, with protein expression patterns closely mirroring those observed following TOP2A silencing (**Figure 9F**). In summary, integrating these experimental findings with earlier bioinformatic analyses, our study functionally establishes that TOP2A critically contributes to HCC progression by modulating DKK1, likely through a β-catenin–mediated EMT mechanism.

### 3.8 Response to TACE, sorafenib and immunotherapy in HCC patients with high TOP2A expression

**Figure 10:**
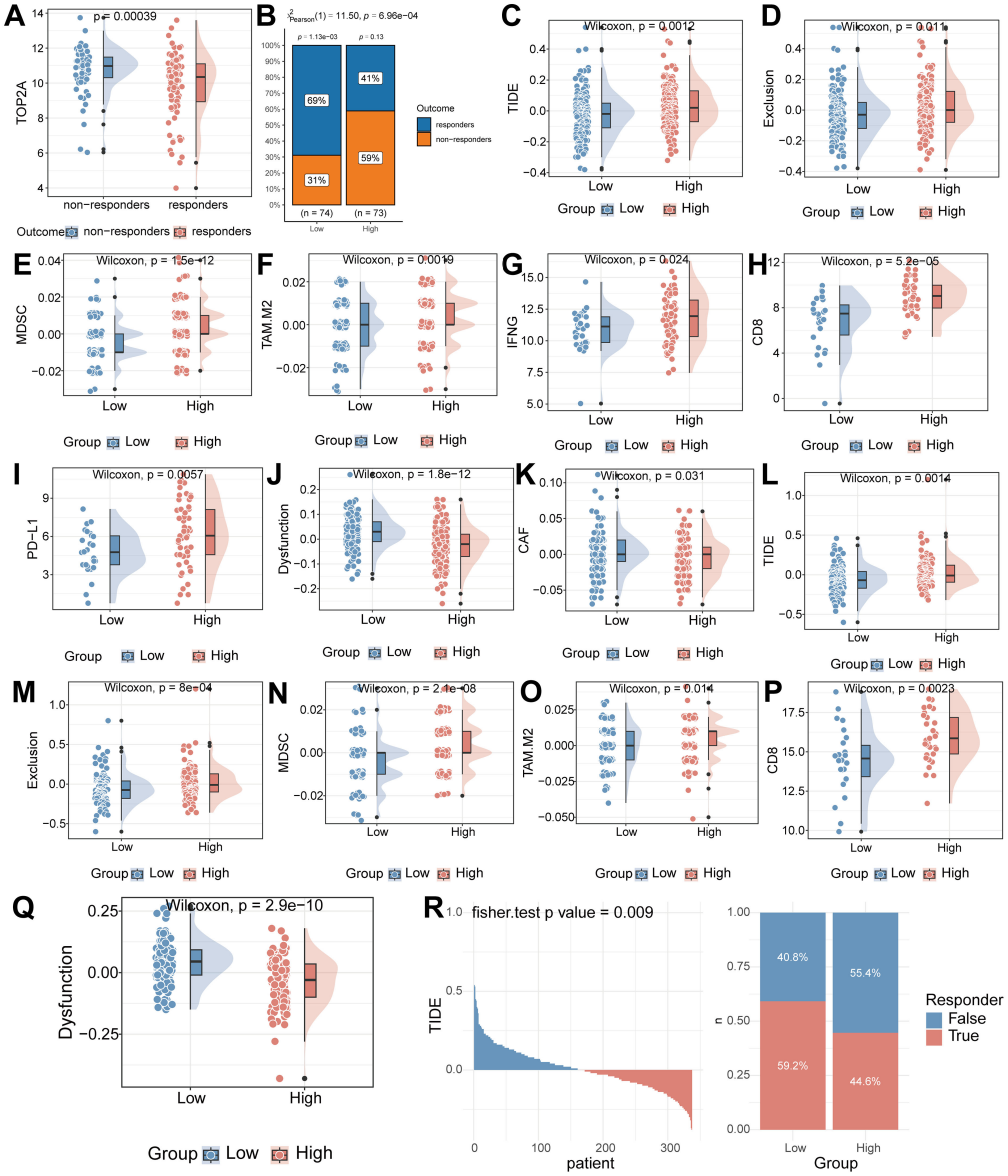
Response to TACE and immunotherapy in patients with high TOP2A expression. **(A)** TOP2A expression in responders versus non-responders to TACE in the GSE104580 dataset. **(B)** Proportion of TACE responders and non-responders stratified by high versus low TOP2A expression in the GSE104580 dataset. **(C–K)** Differences in immune-related scores—including TIDE, Exclusion, MDSC, TAM.M2, IFNG, CD8, PD-L1, Dysfunction, and CAF—between patients with high and low TOP2A expression in the TCGA cohort. **(L–Q)** Differences in immune-related scores—including TIDE, Exclusion, MDSC, TAM.M2, CD8 and Dysfunction—between patients with high and low TOP2A expression in the ICGC cohort. **(R)** Predicted response to immunotherapy between high and low TOP2A expression groups in the TCGA cohort, based on TIDE algorithm analysis.

Transarterial chemoembolization (TACE), targeted therapy, and immunotherapy are pivotal treatment strategies for patients with intermediate to advanced HCC. Given the established role of TOP2A in HCC pathogenesis and its significant impact on patient prognosis, this study further investigated the association between high TOP2A expression and therapeutic efficacy in clinical. Analysis of the GSE104580 dataset, which included 147 patients treated with TACE, revealed that TOP2A expression was significantly lower in TACE responders compared to non-responders (**Figure 10A**). Consistently, the proportion of TACE responders was markedly reduced among patients with high TOP2A expression (69% vs 41%, **Figure 10B**). In the GSE109211 cohort of 67 sorafenib-treated patients (18), TOP2A expression levels were also lower in responders (**Supplementary Figure 8A**), and a lower response ratio was observed in the high TOP2A expression subgroup (34% vs 28%, **Supplementary Figure 8B**), although these differences were not statistically significant.

Additionally, the TIDE algorithm was employed to evaluate the association between TOP2A expression and immunotherapy response in TCGA and ICGC cohorts (37). In the TCGA cohort, the high TOP2A expression group exhibited significantly elevated TIDE scores, immune exclusion scores, and levels of MDSCs, TAM.M2, IFNG, CD8, and PD-L1, alongside lower immune dysfunction scores and CAF levels (p < 0.05; **Figures 10C–K**). This immune profile was largely corroborated in the ICGC cohort, where the high TOP2A group consistently showed higher TIDE scores, immune exclusion scores, and levels of MDSCs, TAM.M2, and CD8, as well as lower immune dysfunction scores (p < 0.05; **Figures 10L–Q**), although differences in IFNG, PD-L1, and CAF levels did not reach statistical significance (**Supplementary Figures 8C–E**). Notably, a higher proportion of patients in the high TOP2A group were predicted to have a poor immunotherapy response than the low-expression group in both the TCGA (55.4% vs. 40.8%, **Figure 10R**) and ICGC (49.6% vs. 37.9%, **Supplementary Figure 7F**) cohorts, although the difference was not statistically significant in the latter. These findings collectively indicate that elevated TOP2A expression may influence the tumor immune microenvironment, which in turn contributes to the diminished efficacy of immunotherapy.

### 3.9 Screening and validation of small-molecule candidate compounds targeting TOP2A

**Figure 11:**
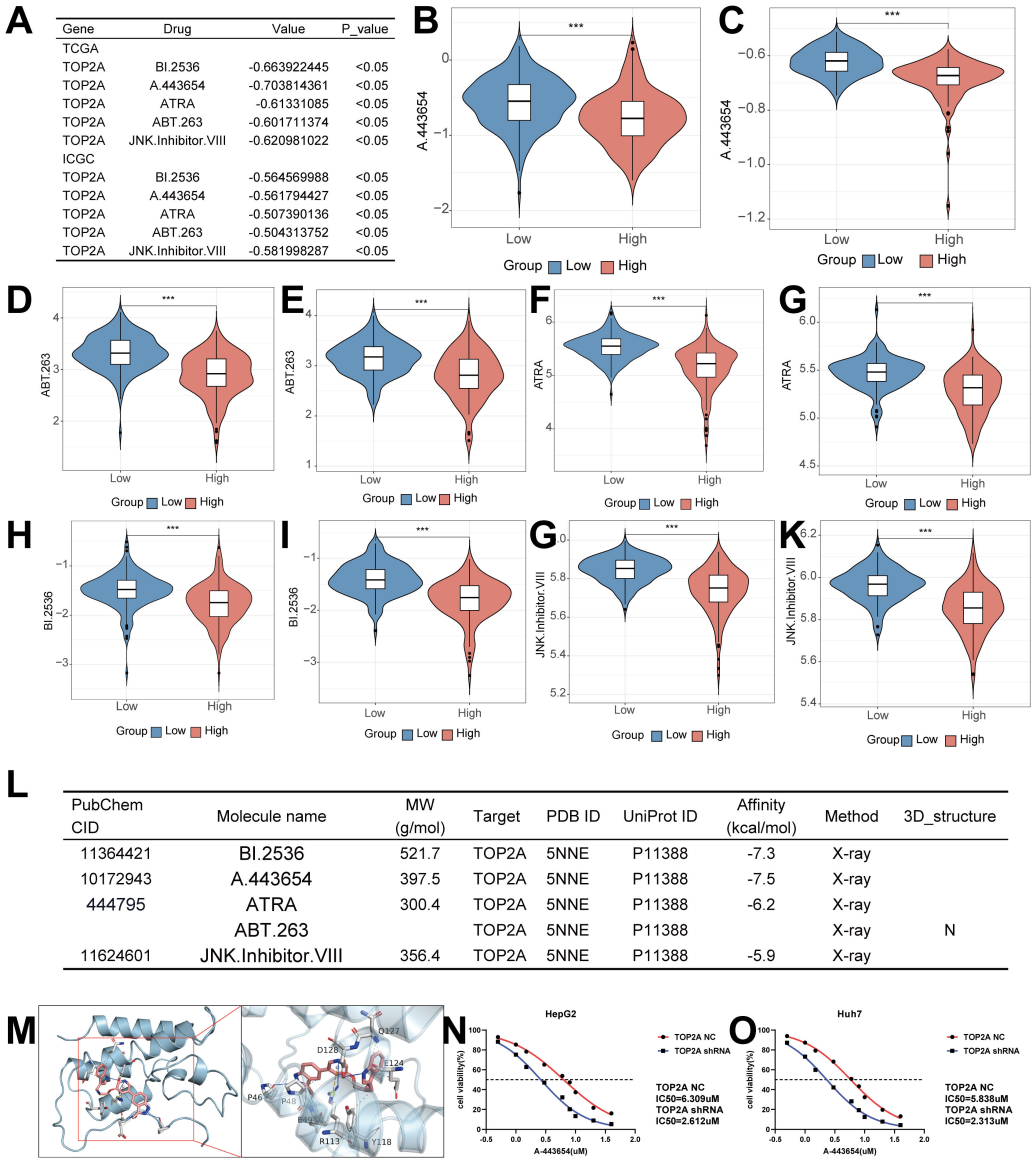
Drug sensitivity and molecular docking analysis targeting TOP2A. **(A)** The correlation between TOP2A expression and drug sensitivity (IC_50_) was analyzed in the TCGA-LIHC and ICGC-HCC cohorts. **(B–K)** Comparison of drug sensitivity (IC_50_ values) for BI.2536 **(B, C)**, A.443654 **(D, E)**, ATRA **(F, G)**, ABT.263 **(H, I)**, and JNK Inhibitor VIII **(G, K)** between TOP2A-high and TOP2A-low groups in the TCGA-LIHC and ICGC-LIHC cohorts. Significance was determined by Wilcoxon rank-sum test; ***p < 0.001. **(L)** Structural and binding characteristics of tested drugs, including PubChem CID, molecular weight (MW), predicted target (TOP2A), PDB ID, UniProt ID, binding affinity (kcal/mol), and key structural features. **(M)** Molecular docking models illustrating the potential interaction between TOP2A and A-443654, highlighting key binding residues (left) and ligand–protein interaction patterns (right). **(N, O)** Dose-response curve of A-443654 in HepG2 cells **(N)** and Huh7 cells **(O)**. Cell viability was compared between TOP2A-knockdown (TOP2A shRNA, blue) and non-targeting control shRNA (TOP2A NC, red) groups. The IC_50_ values were 6.309 μM (NC) and 2.612 μM (shRNA). Data represent the mean ± SD from three independent experiments.

Given the potential impact of high TOP2A expression on HCC patients’ responses to transarterial chemoembolization (TACE), sorafenib, and immunotherapy, pharmacological inhibition of TOP2A could potentially improve patient prognosis. Although TOP2A is a well-established target for multiple anticancer agents, including anthracyclines and epipodophyllotoxins, their clinical utility is often limited by systemic toxicity, drug resistance, and off-target effects (39). To explore drugs closely associated with TOP2A, we employed the pRRopheticR package on TCGA-LIHC and ICGC-HCC datasets to identify compounds whose sensitivity correlates strongly with TOP2A expression levels. Screening analysis identified several small-molecule compounds—including BI.2536, A.443654, ATRA, ABT.263, and JNK.Inhibitor.VIII—whose IC_50_ values showed a strong negative correlation with TOP2A expression in both datasets (r < –0.5, p < 0.05; **Figure 11A**). Accordingly, the high TOP2A expression group exhibited significantly lower IC_50_ values for these agents compared to the low expression group (p < 0.05, **Figures 11B–K**).

Molecular docking analysis further demonstrated that among these compounds, A-443654 exhibited the highest binding affinity for TOP2A, with a binding free energy of -7.5 kcal/mol (**Figure 11L**). The interaction analysis further predicted several potential molecular contacts within the binding pocket: R113 formed a Π-cation interaction, while P48, E49, Y118, E124, and Q127 residues engaged in hydrophobic interactions with A-443654, while residues D128, P46, and R113 were involved in hydrogen bond formation (**Figure 11M**). Meanwhile, in HCC cell lines, knockdown of TOP2A significantly reduced the IC50 value of A-443654 (**Figures 11N, O**), supporting the potential interaction of TOP2A and A-443654. Despite its known role as an Akt inhibitor, A-443654 shows potential interaction with TOP2A in our findings, pending further biophysical validation to confirm direct binding.

### 3.10 TOP2A knockdown suppresses tumor growth *in vivo*

**Figure 12:**
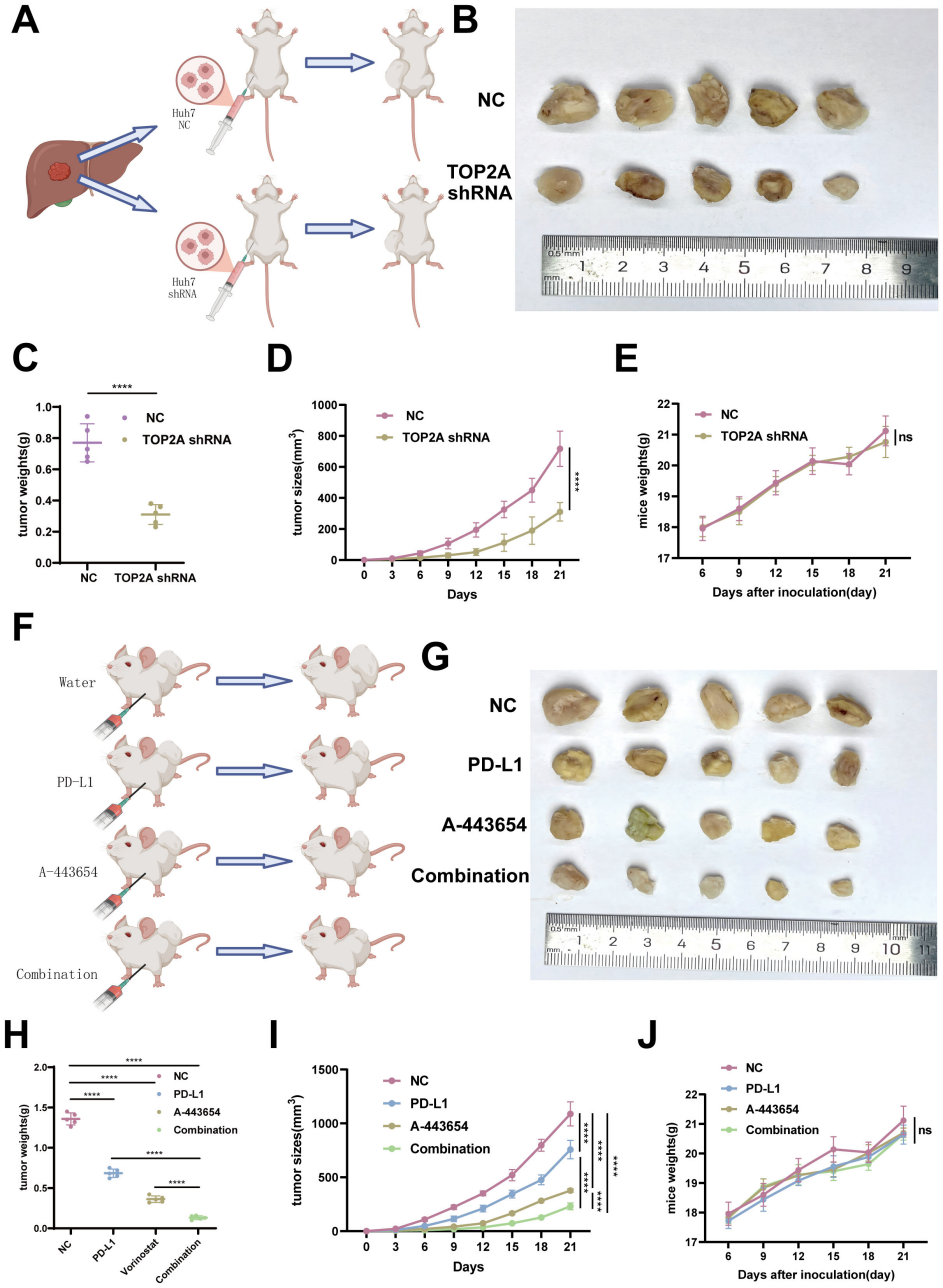
TOP2A promotes hepatocellular carcinoma proliferation *in vivo*. **(A)** Schematic of the mouse xenograft model. **(B)** Representative images of subcutaneous xenograft tumors from each group (n = 5). **(C)** Tumor weights and **(D)** volumes (measured every 3 days) in control and TOP2A-knockdown (shRNA) groups. **(E)** Body weights of mice throughout the experiment. **(F)** Schematic of drug treatment groups in the xenograft model. **(G)** Representative images of tumors from each drug treatment group (n = 5). **(H)** Tumor weights and **(I)** volumes across control and drug-treated groups. **(J)** Body weights of mice in all groups. Data are presented as mean ± SD; **p < 0.01, ***p < 0.001, ****p < 0.0001.

Building on previous findings that established TOP2A as a key molecular driver in HCC, we further investigated its functional role through an *in vivo* xenograft model. Huh7 cells stably expressing TOP2A-specific shRNA or negative control (NC) shRNA were subcutaneously inoculated into nude mice (**Figure 12A**). TOP2A knockdown led to marked suppression of tumor growth, as reflected by significantly reduced tumor weight, attenuated growth kinetics, and stable body weight throughout the experiment (**Figures 12B–E**). Furthermore, prompted by our earlier observation that TOP2A expression positively correlates with PD-L1 and contributes to a dysregulated immune microenvironment, we evaluated the therapeutic potential of combining TOP2A inhibition with immune checkpoint blockade. In a syngeneic preclinical model, Hepa 1–6 cells were injected subcutaneously into C57BL/6 mice, which were allocated into four groups: control, TOP2A candidate inhibitor (A-443654) monotherapy, anti–PD-L1 monotherapy, and combination therapy (**Figure 12F**). The combined regimen elicited significantly greater tumor suppression than either agent alone, underscoring a synergistic antitumor interaction between TOP2A candidate inhibitor and PD-L1 blockade (**Figures 12G–I**). No significant body weight variations were detected among the groups, suggesting a favorable safety profile (**Figure 12J**).

## 4 Discussion

Hepatocellular carcinoma (HCC) remains a malignancy of high incidence and poor prognosis (3), necessitating continued investigation into its molecular drivers and therapeutic vulnerabilities. Building upon the established role of TOP2A in cancer biology (40–42), our study comprehensively validates the clinical and functional significance of TOP2A in HCC. TCGA pan-cancer analysis showed consistently elevated TOP2A expression, with marked upregulation in HCC confirmed at the protein level. TOP2A showed high diagnostic accuracy (AUC = 0.935) and correlated with advanced tumor stage. Importantly, elevated TOP2A expression predicted poorer OS in both TCGA-LIHC and ICGC-HCC cohorts, affirming its broad prognostic relevance. Immunohistochemical analysis in our institutional cohort further demonstrated that high TOP2A protein expression was independently associated with reduced survival (HR = 1.014, 95% CI: 1.007–1.020). Functionally, TOP2A knockdown consistently suppressed proliferation, clonogenicity, migration, and invasion, supporting its role as a key oncogenic driver in HCC. These findings—spanning bioinformatic analysis, clinical validation, and functional experiments—strongly establish TOP2A as a promising biomarker and potential therapeutic target in HCC.

Building upon TOP2A’s established significance, we further delineated its regulatory context by identifying E2F6 as a novel upstream transcriptional regulator. E2F6, a key member of the E2F transcription factor family, is crucial for regulating eukaryotic cell proliferation and has been increasingly implicated in cancer progression (43). In the context of HCC, emerging evidence has begun to elucidate E2F6’s multifaceted roles in driving tumor progression (44). Hou et al. demonstrated that E2F6 directly binds to the UPF3B promoter, enhancing its transcription and subsequently activating the PI3K/AKT/mTOR pathway to promote HCC growth (45). Similarly, Liu et al. revealed that CENPU interacts with E2F6, promoting its ubiquitination and degradation, which in turn influences E2F1 transcription and accelerates the G1-to-S phase transition, thereby enhancing HCC cell proliferation (44). These studies collectively establish E2F6 as a significant player in HCC pathogenesis, though its complete regulatory network remains to be fully elucidated.

Through comprehensive bioinformatics analysis using the LncMAP web tool (24) and JASPAR database (26), we identified E2F6 as a prime candidate for TOP2A regulation, supported by its significant dysregulation in HCC tissues, strong prognostic value, and well-documented transcription factor binding sites. Consistent with previous reports (46), our analysis confirmed significantly elevated E2F6 expression in HCC tissues, with high expression levels correlating with poor prognosis. The robust positive correlation between E2F6 and TOP2A expression across both TCGA and ICGC cohorts (TCGA: R = 0.73; ICGC: R = 0.66) provided initial evidence for their potential regulatory relationship. This was further substantiated by functional experiments demonstrating that E2F6 knockdown significantly reduced TOP2A expression at both mRNA and protein levels, while TOP2A knockdown did not affect E2F6 expression, confirming a unidirectional regulatory relationship. Most importantly, ChIP-PCR confirmed that E2F6 directly binds to the TOP2A promoter, thereby regulating its transcription.

The clinical relevance of this regulatory relationship is underscored by several key observations. First, E2F6 itself demonstrates significant diagnostic and prognostic value in HCC, with elevated expression in tumor tissues, increasing expression with advancing tumor stage, and strong association with poor overall survival—patterns that closely mirror those observed for TOP2A. Second, patients exhibiting concurrent high expression of both E2F6 and TOP2A (E2F6_H&TOP2A_H) demonstrated significantly worse prognosis, highlighting the clinical importance of this molecular subgroup. The predictive model we developed, incorporating both E2F6 and TOP2A expression levels, showed exceptional performance in identifying these high-risk patients across multiple validation cohorts (AUC values: TCGA: 0.989; ICGC: 0.985; GEO datasets: 0.810-0.937), demonstrating robust generalizability and clinical applicability.

Functionally, the E2F6-TOP2A axis appears to drive HCC progression through multiple mechanisms. Differential gene expression analysis revealed that the E2F6_H&TOP2A_H subgroup exhibits significant enrichment in processes related to epithelial-mesenchymal transition (EMT), cell adhesion, extracellular matrix organization, and proliferative pathways. The strong positive correlation between both E2F6 and TOP2A expression levels and EMT activation further supports the conclusion that E2F6 promotes HCC progression through transcriptional upregulation of TOP2A, which in turn drives EMT and metastatic processes. These findings align with and substantially expand upon previous studies documenting E2F6’s involvement in HCC pathogenesis by establishing its direct regulatory relationship with TOP2A as a novel mechanism through which E2F6 exerts its oncogenic functions (46).

To complete the molecular mechanism, we investigated TOP2A’s downstream genes and identified DKK1 as a key mediator of its oncogenic functions. DKK1, a secreted modulator of Wnt/β-catenin signaling, demonstrates context-dependent roles in cancer (38). While it acts as a tumor suppressor in colorectal cancer (47–49), DKK1 exhibits oncogenic properties in multiple malignancies including NSCLC, SCLC, esophageal carcinoma (50), breast cancer, and kidney cancer (51). Consistent with prior reports in HCC (38), our multi-algorithm bioinformatic analysis consistently identified DKK1 upregulation in TOP2A-high HCC samples, with significant enrichment of EMT and Wnt/β-catenin pathways. Functional experiments demonstrated that DKK1 knockdown reversed TOP2A-driven malignant phenotypes, establishing DKK1 as an essential mediator of TOP2A’s oncogenic effects. To further investigate whether DKK1 regulates β-catenin signaling in HCC cells, immunofluorescence analysis was performed to assess its subcellular localization. The results showed that DKK1 knockdown reduced cytoplasmic β-catenin levels and nuclear accumulation, whereas DKK1 overexpression promoted cytoplasmic accumulation and enhanced nuclear translocation of β-catenin in Huh7 and HepG2 cells. Mechanistically, our results suggest that TOP2A promotes EMT progression through DKK1-associated regulation of β-catenin signaling. Both TOP2A silencing and DKK1 knockdown led to reversal of EMT marker expression and suppression of β-catenin levels, collectively suggesting a regulatory TOP2A–DKK1–β-catenin–EMT axis in HCC progression.

In a word, our study comprehensively characterizes the E2F6-TOP2A-DKK1 regulatory axis, demonstrating how E2F6-driven TOP2A transcription upregulates DKK1 to drive EMT and metastasis via β-catenin signaling. These findings advance our understanding of HCC pathogenesis and reveal multiple therapeutic targeting opportunities within this signaling cascade. Beyond the core signaling axis, we explored the therapeutic implications of TOP2A expression in HCC. Our study demonstrated that elevated TOP2A expression correlates with advanced tumor stage and predicted poor response to TACE and reduced sensitivity to sorafenib. Analysis using the TIDE algorithm revealed that TOP2A-high tumors exhibit a complex immune microenvironment: despite showing elevated CD8—reflecting active immune infiltration—these tumors also display increased immune exclusion, higher infiltration of MDSCs and TAM.M2 cells, and elevated TIDE scores. This is consistent with prior studies demonstrating that MDSCs and M2-polarized tumor-associated macrophages can physically restrict cytotoxic T cells from entering the tumor core, creating an immune-excluded microenvironment that limits immunotherapy efficacy (52–54). In this context, the accumulation of MDSCs and TAM.M2 in TOP2A-high tumors may similarly sequester infiltrating T cells at the tumor periphery, thereby promoting immune evasion and diminishing the efficacy of immune checkpoint inhibitors. Our findings further underscore the complexity of the tumor immune microenvironment, as reported in recent studies (55, 56).

To overcome this therapeutic resistance, we selected A-443654 as a candidate compound for further investigation. A-443654 was originally identified as a pan-AKT inhibitor and has been shown to potently suppress AKT signaling across multiple cancer types (57). Notably, several independent computational studies have predicted A-443654 sensitivity in HCC (58–60), although experimental validation remains largely lacking. Consistent with these predictions, our pRRophetic analysis and molecular docking further supported a potential interaction between A-443654 and TOP2A, providing a rationale for subsequent functional validation. TOP2A knockdown sensitized HCC cells to A-443654, lowering its IC50, suggesting that A-443654 efficacy may be partially dependent on TOP2A expression. *In vivo*, both A-443654 and a PD-L1 inhibitor monotherapies suppressed tumor growth, while their combination resulted in synergistic antitumor effects. These findings support a novel combinatory strategy to overcome immunotherapy resistance in TOP2A-high HCC.

While our study comprehensively characterized the E2F6-TOP2A-DKK1 regulatory axis in HCC, several limitations should be acknowledged. First, while HepG2 cells were used for mechanistic assays, their hepatoblastoma-like background is a known limitation in HCC research. To address this, we verified our core functional findings in authentic HCC cell lines (Huh7 and HCCLM3). Second, the clinical correlations regarding treatment response primarily derive from retrospective analyses of public datasets, necessitating validation in prospective clinical cohorts. Third, although we established the direct regulatory relationship between E2F6 and TOP2A, the precise molecular mechanisms governing E2F6 expression and activity in HCC require further investigation. Fourth, while our functional experiments demonstrated the essential role of DKK1 in mediating TOP2A’s oncogenic effects, the complete repertoire of TOP2A-regulated effectors likely extends beyond DKK1 alone. Finally, the translational potential of the A-443654 and anti-PD-L1 combination therapy requires more comprehensive evaluation, including the validation of the binding interaction between TOP2A and A-443654, as well as of potential toxicity and resistance mechanisms.

In summary, our study systematically explored the E2F6-TOP2A-DKK1 regulatory axis as a key driver of HCC progression, spanning from transcriptional regulation to downstream effector mechanisms. We demonstrated how E2F6-driven TOP2A transcription upregulates DKK1 to activate β-catenin signaling and EMT, ultimately promoting metastasis and therapeutic resistance. The development of robust predictive models for patient stratification, combined with the identification of a novel combination therapy strategy, provided a strong foundation for future clinical translation. These findings substantially advanced our understanding of HCC molecular pathogenesis and offer multiple therapeutic targeting opportunities within this signaling cascade, potentially improving outcomes for HCC patients characterized by high TOP2A expression.

## 5 Conclusions

Our study identifies the E2F6–TOP2A–DKK1 axis as a key regulatory pathway driving hepatocellular carcinoma (HCC) progression. We demonstrate that E2F6 directly activates TOP2A transcription, and the resulting TOP2A upregulation promotes epithelial–mesenchymal transition (EMT) and metastatic dissemination by inducing DKK1 expression and activating β-catenin signaling. Although the HepG2 cell line has recognized limitations, these conclusions are firmly supported by convergent evidence from our comprehensive validation using two additional authentic HCC models. Critically, we constructed a combined E2F6-TOP2A signature that effectively identifies a patient subgroup with co-upregulation of both markers, which is associated with the poorest clinical outcomes. Moreover, given that high TOP2A expression contributes to resistance to TACE, sorafenib, and immune checkpoint blockade, we propose a therapeutic strategy combining the AKT inhibitor A-443654 with anti-PD-L1 therapy. This combinatorial approach provides mechanistic rationale and preclinical evidence for overcoming therapy resistance in TOP2A-high HCC.

## Data Availability

The datasets presented in this study can be found in online repositories. The names of the repository/repositories and accession number(s) can be found in the article/**Supplementary Material**.

## Supplementary material

Supplementary Table 1 Clinical information of different database cohorts.

Supplementary Table 2 The shRNA sequences of all primers.

Supplementary Table 3 Primers for ChIP-qPCR.

Supplementary Table 4 Deg between the E2F6_H&TOP2A_H and the others.

Supplementary Table 5 GO enrichment analysis of DEGs.

Supplementary Table 6 KEGG enrichment analysis of DEGs.

Supplementary Figure 1 TOP2A demonstrates diagnostic and prognostic value in HCC based on the ICGC-HCC cohort. **(A)** TOP2A mRNA expression is significantly upregulated in HCC compared with non-tumor tissues in the ICGC dataset (p < 0.05). **(B)** ROC analysis confirms the high diagnostic accuracy of TOP2A expression in discriminating HCC from non-tumor samples (AUC = 0.956, p < 0.05). **(C)** The TOP2A expression shows no statistically significant variation across the HCC clinical stages (p = 0.17, Kruskal–Wallis test). **(D)** High TOP2A expression is associated with significantly poorer overall survival in HCC patients (p < 0.05).

Supplementary Figure 2 TOP2A knockdown suppresses malignant phenotypes in HCCLM3 cells. **(A)** Validation of TOP2A knockdown efficiency in HCCLM3 cells by RT-qPCR. **(B)** CCK-8 assay showing the effect of TOP2A silencing on cell proliferation over time (0–4 days). **(C)** Colony formation assay demonstrating reduced clonogenic ability following TOP2A knockdown; representative images and quantitative analysis are shown. **(D)** Transwell migration and invasion assays showing that TOP2A depletion significantly inhibits migratory and invasive capacities of HCCLM3 cells; representative images and corresponding quantification are presented. **(E)** Wound healing assay (0 h and 24 h) illustrating impaired cell migration after TOP2A knockdown; quantification of migrated distance is shown on the right. Data are presented as *mean ± SD.* *P < 0.05, **P < 0.01, ***P < 0.001, ****P < 0.0001.

Supplementary Figure 3 Diagnostic and prognostic significance of E2F6 in HCC (ICGC-HCC cohort). **(A)** Elevated E2F6 expression in tumor tissues compared with non-tumor tissues in the ICGC-HCC dataset (p < 0.05, Wilcoxon test). **(B)** Receiver operating characteristic (ROC) curve demonstrating the diagnostic performance of E2F6 in discriminating HCC from non-tumor samples (AUC = 0.828, p < 0.05). **(C)** E2F6 expression across clinical stages (I, II, and III/IV) of HCC from the ICGC cohort (Kruskal–Wallis test; p < 0.05). **(D)** Kaplan–Meier analysis confirming poorer overall survival in patients with high E2F6 expression (p < 0.05).

Supplementary Figure 4 Validation and clinical utility of the predictive model in independent cohorts. **(A)** ROC analysis of the combined model in the ICGC-HCC cohort, demonstrating excellent discriminative ability (AUC = 0.985, p < 0.05). **(B)** ROC analysis of the combined model in the GSE109211 cohort, showing moderate yet significant discriminative ability (AUC = 0.810, p < 0.05). **(C)** ROC analysis of the combined model in the GSE104580 cohort, confirming strong predictive performance (AUC = 0.937, p < 0.05). **(D)** Decision curve analysis (DCA) for the ICGC-HCC cohort, revealing superior net benefit of the combined model (red) over individual markers (E2F6 in green, TOP2A in blue) and the full feature set (dashed lines). **(E)** DCA for the GSE109211 cohort, further supporting the enhanced clinical utility of the combined model across risk thresholds. **(F)** DCA for the GSE104580 cohort, consistently demonstrating the optimal net benefit of the combined model in patient stratification. Note: p-values denote statistical significance; all analyses incorporated appropriate multiple testing corrections.

Supplementary Figure 5 Validation of DKK1 and TOP2A overexpression and knockdown in HCC cells. (A-B) DKK1 mRNA and protein expression in HepG2 and Huh7 cells transfected with DKK1 PCDH or DKK1 shRNA, measured by RT-qPCR and Western blotting. (C-D) TOP2A mRNA and protein expression in HepG2 and Huh7 cells transfected with TOP2A PCDH or TOP2A OE, analyzed by RT-qPCR and Western blotting.

Supplementary Figure 6 DKK1 is required for TOP2A-induced malignant phenotypes in HCCLM3 cells. **(A)** CCK-8 assay showing the effect of TOP2A overexpression (TOP2A OE) and DKK1 knockdown (TOP2A OE + DKK1 shRNA) on the proliferation of HCCLM3 cells compared with control (TOP2A NC). **(B, C)** Colony formation assay demonstrating that TOP2A overexpression significantly enhances clonogenic ability, which is markedly attenuated by DKK1 knockdown. Quantification of colony numbers is shown in **(C, D)** Transwell migration and invasion assays showing that TOP2A overexpression promotes migratory and invasive capacities of HCCLM3 cells, while DKK1 knockdown significantly reverses these effects. Representative images are shown on the left, and quantitative analysis is shown on the right. Scale bar = 50 μm. **(E)** Wound healing assay indicating that TOP2A overexpression accelerates wound closure in HCCLM3 cells, whereas DKK1 knockdown suppresses this effect. Representative images at 0 h and 24 h are shown on the left, with quantitative analysis of migrated distance shown on the right. Data are presented as *mean ± SD*. Statistical significance was determined using appropriate tests (*P < 0.05, **P < 0.01, ***P < 0.001, ****P < 0.0001).

Supplementary Figure 7 DKK1 regulates β-catenin subcellular localization in HCC cells. **(A)** Huh7 cells and **(B)** HepG2 cells were subjected to DKK1 knockdown (DKK1 shRNA), negative control (DKK1 NC), or DKK1 overexpression (DKK1 OE). β-catenin was detected by immunofluorescence (green), and nuclei were counterstained with DAPI (blue). Merged images are shown in the right panels. Representative images indicate that modulation of DKK1 expression alters the distribution pattern of β-catenin in both cell lines. Scale bar, 10 μm.

Supplementary Figure 8 Association of TOP2A expression with response to sorafenib and immunotherapy. **(A)** TOP2A expression levels in sorafenib responders versus non-responders within the GSE109211 dataset. **(B)** Proportion of sorafenib responders stratified by high and low TOP2A expression in the GSE109211 cohort. **(C-E)** Differences in immune-related scores—including IFNG, PD-L1 and CAFs—between patients with high and low TOP2A expression in the ICGC cohort. **(F)** Predicted response to immunotherapy between high and low TOP2A expression groups in the ICGC cohort, based on TIDE algorithm analysis.
