# Supplementary material for: Targeting the E2F6-TOP2A-DKK1 axis: a novel therapeutic strategy for EMT-driven hepatocellular carcinoma progression
Source: Front Immunol. 2026 Jul 2;17:1809952. doi: 10.3389/fimmu.2026.1809952 (PMC13373062; doi:10.3389/fimmu.2026.1809952)
Supplement: Supplementary Table 2 — The shRNA sequences of all primers. [file Table2.docx]

Table S1 Clinical information of different database cohort.

|  | TCGA |  | ICGC |  | GSE109211 |  | GSE104580 |  | Total |
| --- | --- | --- | --- | --- | --- | --- | --- | --- | --- |
|  | (N=337) |  | (N=231) |  | (N=140) |  | (N=147) |  | (N=855) |
| Age |  |  |  |  |  |  |  |  |  |
| Mean (SD) | 59.9 (13.3) |  | 67.3 (10.1) |  | NA |  | NA |  | 62.9 (12.6) |
| Median  [Min, Max] | 62.0  [17.0, 86.0] |  | 69.0  [31.0, 89.0] |  | NA |  | NA |  | 65.0  [17.0, 89.0] |
| Missing | 0 (0%) |  | 0 (0%) |  | 140 (100%) |  | 147 (100%) |  | 287 (33.6%) |
| Gender |  |  |  |  |  |  |  |  |  |
| Male | 230 (68.2%) |  | 170 (73.6%) |  | 0 (0%) |  | 0 (0%) |  | 400 (46.8%) |
| Female | 107 (31.8%) |  | 61 (26.4%) |  | 0 (0%) |  | 0 (0%) |  | 168 (19.6%) |
| Missing | 0 (0%) |  | 0 (0%) |  | 140 (100%) |  | 147 (100%) |  | 287 (33.6%) |
| Stage |  |  |  |  |  |  |  |  |  |
| I | 168 (49.9%) |  | 36 (15.6%) |  | 0 (0%) |  | 0 (0%) |  | 204 (23.9%) |
| II | 82 (24.3%) |  | 105 (45.5%) |  | 0 (0%) |  | 0 (0%) |  | 187 (21.9%) |
| III | 83 (24.6%) |  | 71 (30.7%) |  | 0 (0%) |  | 0 (0%) |  | 154 (18.0%) |
| IV | 4 (1.2%) |  | 19 (8.2%) |  | 0 (0%) |  | 0 (0%) |  | 23 (2.7%) |
| Missing | 0 (0%) |  | 0 (0%) |  | 140 (100%) |  | 147 (100%) |  | 287 (33.6%) |
| Event |  |  |  |  |  |  |  |  |  |
| Alive | 223 (66.2%) |  | 189 (81.8%) |  | 0 (0%) |  | 0 (0%) |  | 412 (48.2%) |
| Death | 114 (33.8%) |  | 42 (18.2%) |  | 0 (0%) |  | 0 (0%) |  | 156 (18.2%) |
| Missing | 0 (0%) |  | 0 (0%) |  | 140 (100%) |  | 147 (100%) |  | 287 (33.6%) |
| Time |  |  |  |  |  |  |  |  |  |
| Mean (SD) | 27.5 (24.7) |  | 27.1 (14.0) |  | NA |  | NA |  | 27.3 (21.0) |
| Median  [Min, Max] | 19.6  [0.200, 123] |  | 26.0  [0.333, 72.0] |  | NA |  | NA |  | 22.0  [0.200, 123] |
| Missing | 0 (0%) |  | 0 (0%) |  | 140 (100%) |  | 147 (100%) |  | 287 (33.6%) |
| Treatment |  |  |  |  |  |  |  |  |  |
| Plac | 0 (0%) |  | 0 (0%) |  | 73 (52.1%) |  | 0 (0%) |  | 73 (8.5%) |
| Sor | 0 (0%) |  | 0 (0%) |  | 67 (47.9%) |  | 0 (0%) |  | 67 (7.8%) |
| TACE | 0 (0%) |  | 0 (0%) |  | 0 (0%) |  | 147 (100%) |  | 147 (17.2%) |
| Missing | 337 (100%) |  | 231 (100%) |  | 0 (0%) |  | 0 (0%) |  | 568 (66.4%) |
| Outcome |  |  |  |  |  |  |  |  |  |
| non-responders | 0 (0%) |  | 0 (0%) |  | 98 (70.0%) |  | 66 (44.9%) |  | 164 (19.2%) |
| responders | 0 (0%) |  | 0 (0%) |  | 42 (30.0%) |  | 81 (55.1%) |  | 123 (14.4%) |
| Missing | 337 (100%) |  | 231 (100%) |  | 0 (0%) |  | 0 (0%) |  | 568 (66.4%) |
