# Supplementary figures and images for: Targeting the E2F6-TOP2A-DKK1 axis: a novel therapeutic strategy for EMT-driven hepatocellular carcinoma progression
Source: Front Immunol. 2026 Jul 2;17:1809952. doi: 10.3389/fimmu.2026.1809952 (PMC13373062; doi:10.3389/fimmu.2026.1809952)

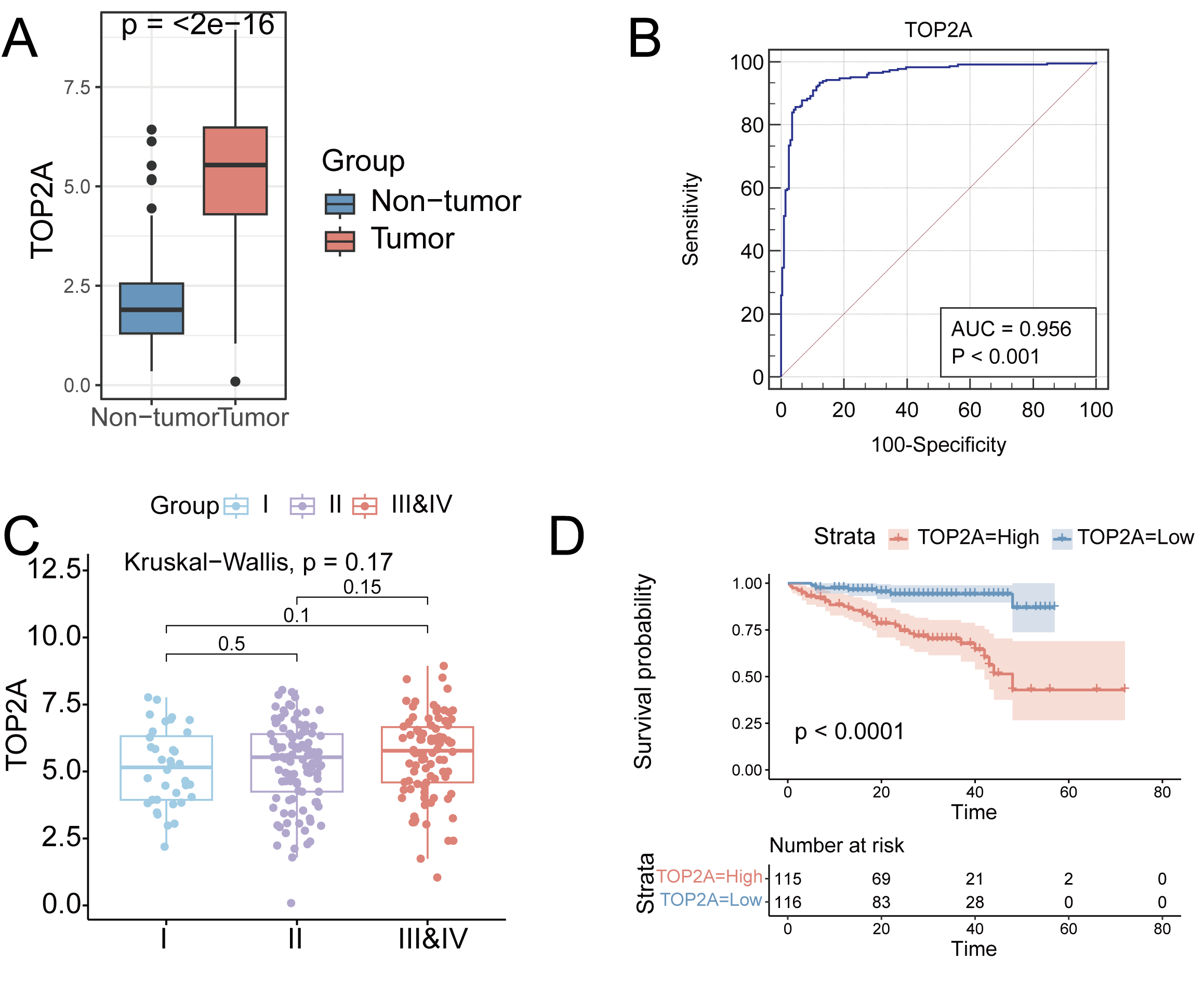

Supplement: Supplementary Figure 1 — TOP2A demonstrates diagnostic and prognostic value in HCC based on the ICGC-HCC cohort. (A) TOP2A mRNA expression is significantly upregulated in HCC compared with non-tumor tissues in the ICGC dataset (p < 0.05). (B) ROC analysis confirms the high diagnostic accuracy of TOP2A expression in discriminating HCC from non-tumor samples (AUC = 0.956, p < 0.05). (C) The TOP2A expression shows no statistically significant variation across the HCC clinical stages (p = 0.17, Kruskal–Wallis test). (D) High TOP2A expression is associated with significantly poorer overall survival in HCC patients (p < 0.05). [file Image1.tif]

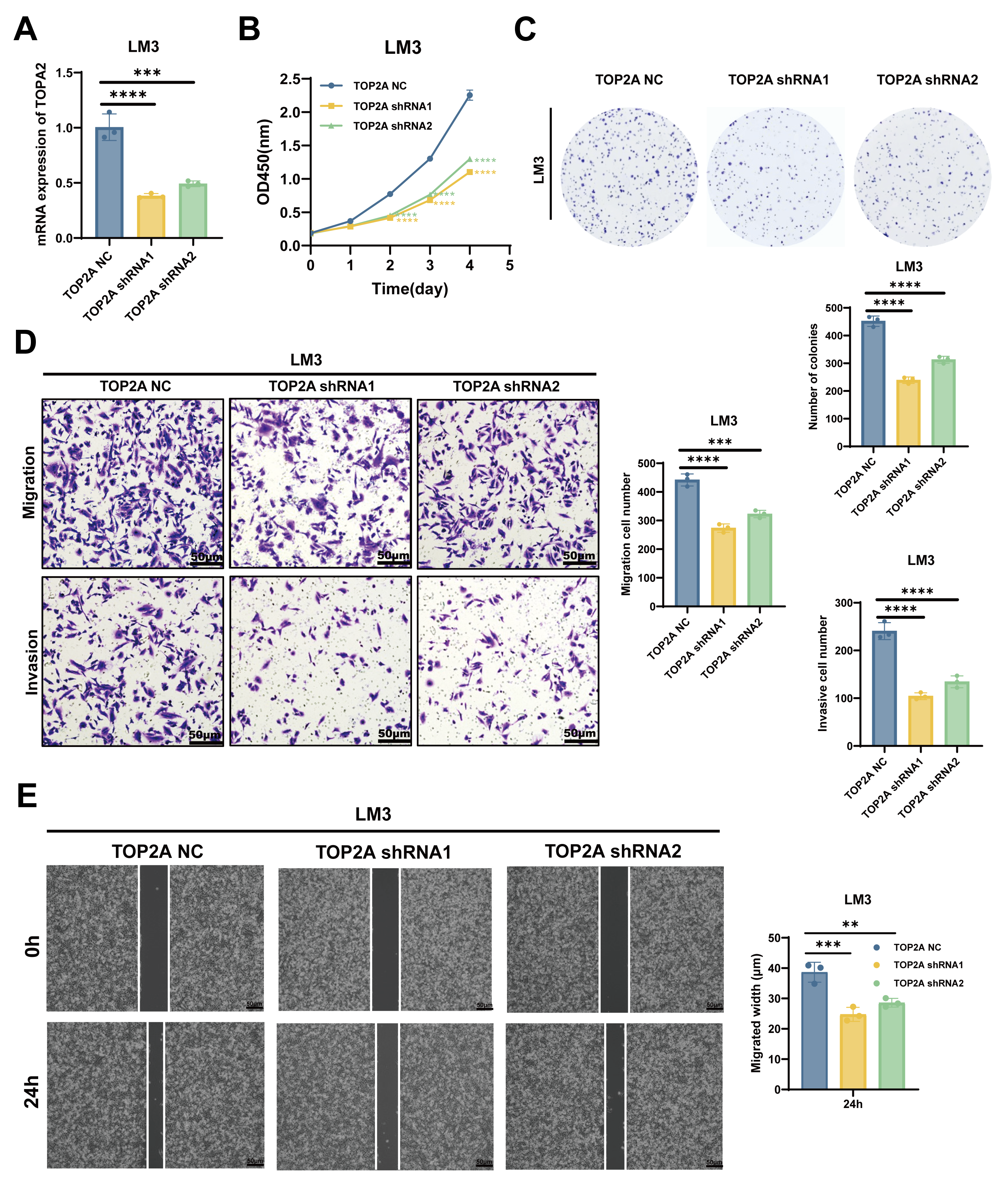

Supplement: Supplementary Figure 2 — TOP2A knockdown suppresses malignant phenotypes in HCCLM3 cells. (A) Validation of TOP2A knockdown efficiency in HCCLM3 cells by RT-qPCR. (B) CCK-8 assay showing the effect of TOP2A silencing on cell proliferation over time (0–4 days). (C) Colony formation assay demonstrating reduced clonogenic ability following TOP2A knockdown; representative images and quantitative analysis are shown. (D) Transwell migration and invasion assays showing that TOP2A depletion significantly inhibits migratory and invasive capacities of HCCLM3 cells; representative images and corresponding quantification are presented. (E) Wound healing assay (0 h and 24 h) illustrating impaired cell migration after TOP2A knockdown; quantification of migrated distance is shown on the right. Data are presented as mean ± SD. *P < 0.05, **P < 0.01, ***P < 0.001, ****P < 0.0001. [file Image2.tif]

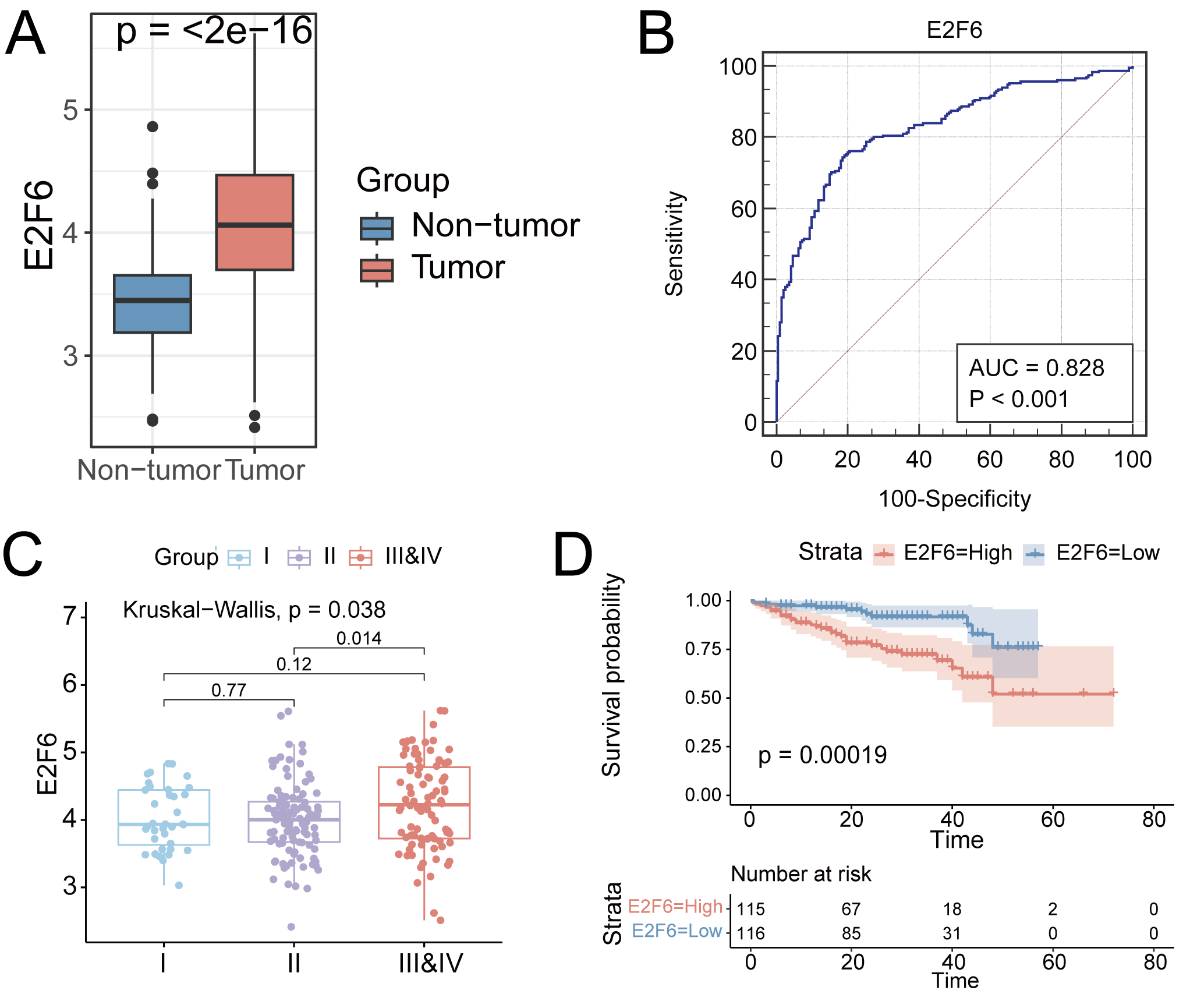

Supplement: Supplementary Figure 3 — Diagnostic and prognostic significance of E2F6 in HCC (ICGC-HCC cohort). (A) Elevated E2F6 expression in tumor tissues compared with non-tumor tissues in the ICGC-HCC dataset (p < 0.05, Wilcoxon test). (B) Receiver operating characteristic (ROC) curve demonstrating the diagnostic performance of E2F6 in discriminating HCC from non-tumor samples (AUC = 0.828, p < 0.05). (C) E2F6 expression across clinical stages (I, II, and III/IV) of HCC from the ICGC cohort (Kruskal–Wallis test; p < 0.05). (D) Kaplan–Meier analysis confirming poorer overall survival in patients with high E2F6 expression (p < 0.05). [file Image3.tif]

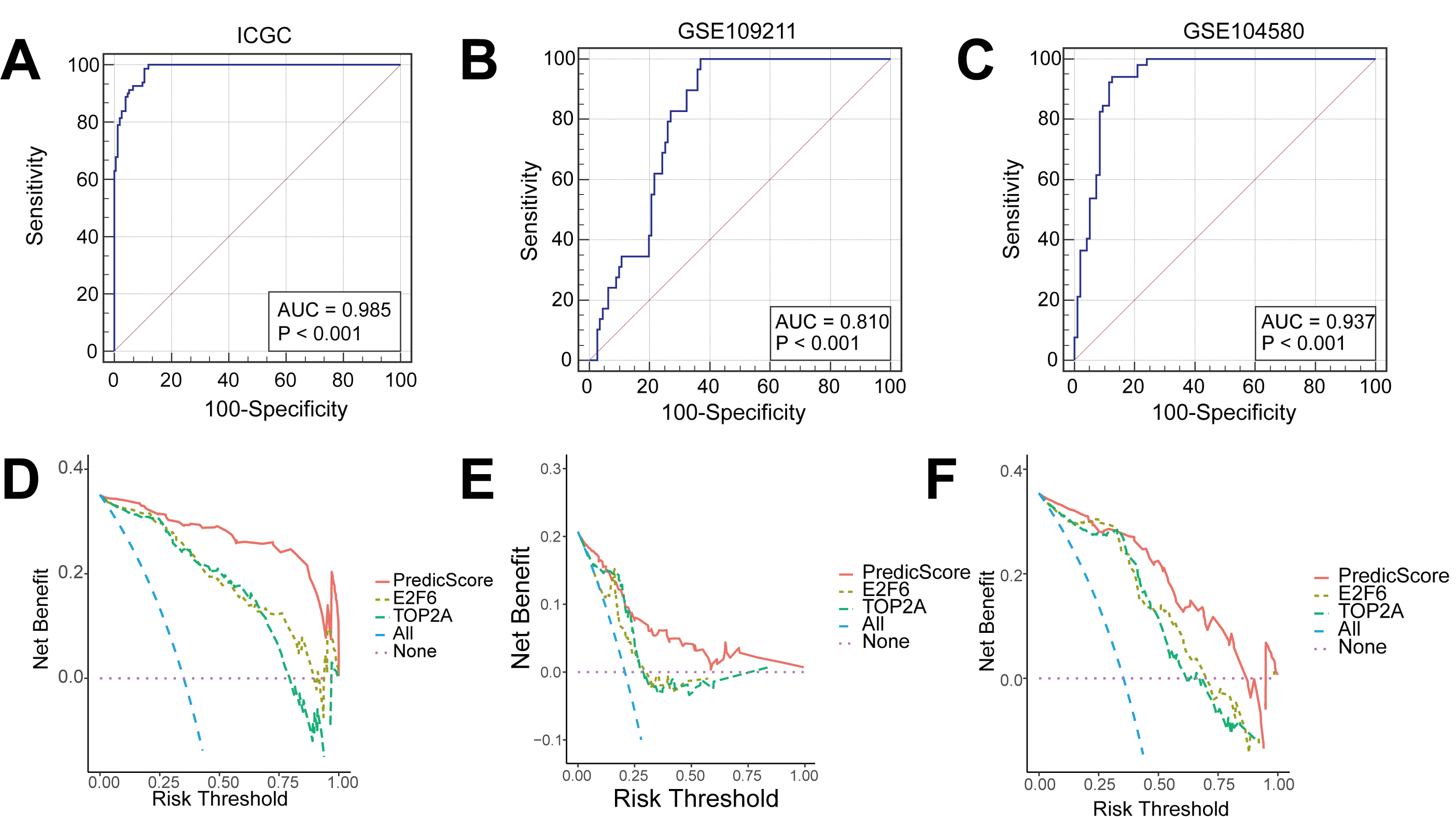

Supplement: Supplementary Figure 4 — Validation and clinical utility of the predictive model in independent cohorts. (A) ROC analysis of the combined model in the ICGC-HCC cohort, demonstrating excellent discriminative ability (AUC = 0.985, p < 0.05). (B) ROC analysis of the combined model in the GSE109211 cohort, showing moderate yet significant discriminative ability (AUC = 0.810, p < 0.05). (C) ROC analysis of the combined model in the GSE104580 cohort, confirming strong predictive performance (AUC = 0.937, p < 0.05). (D) Decision curve analysis (DCA) for the ICGC-HCC cohort, revealing superior net benefit of the combined model (red) over individual markers (E2F6 in green, TOP2A in blue) and the full feature set (dashed lines). (E) DCA for the GSE109211 cohort, further supporting the enhanced clinical utility of the combined model across risk thresholds. (F) DCA for the GSE104580 cohort, consistently demonstrating the optimal net benefit of the combined model in patient stratification. Note: p-values denote statistical significance; all analyses incorporated appropriate multiple testing corrections. [file Image4.tif]

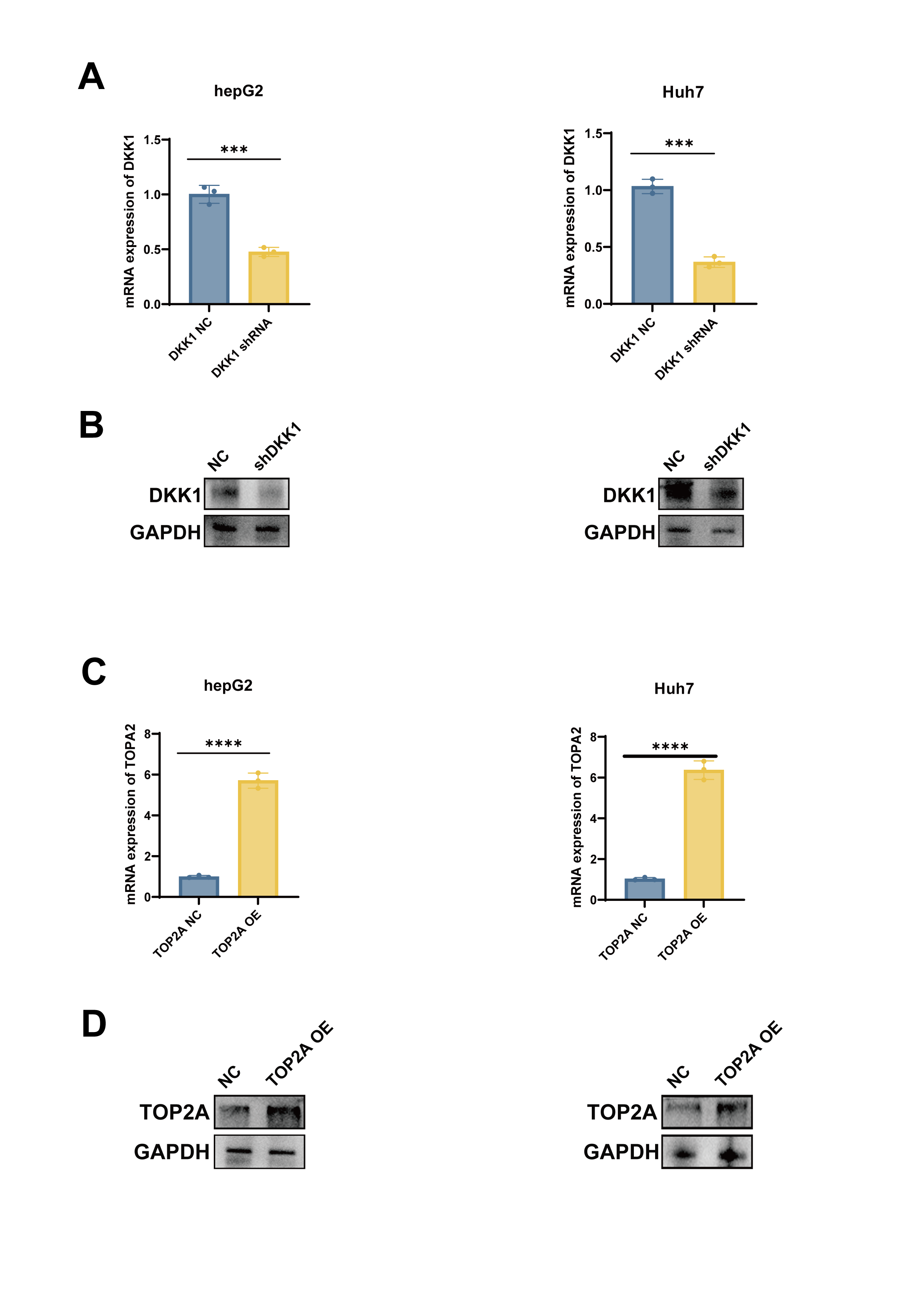

Supplement: Supplementary Figure 5 — Validation of DKK1 and TOP2A overexpression and knockdown in HCC cells. (A-B) DKK1 mRNA and protein expression in HepG2 and Huh7 cells transfected with DKK1 PCDH or DKK1 shRNA, measured by RT-qPCR and Western blotting. (C-D) TOP2A mRNA and protein expression in HepG2 and Huh7 cells transfected with TOP2A PCDH or TOP2A OE, analyzed by RT-qPCR and Western blotting. [file Image5.tif]

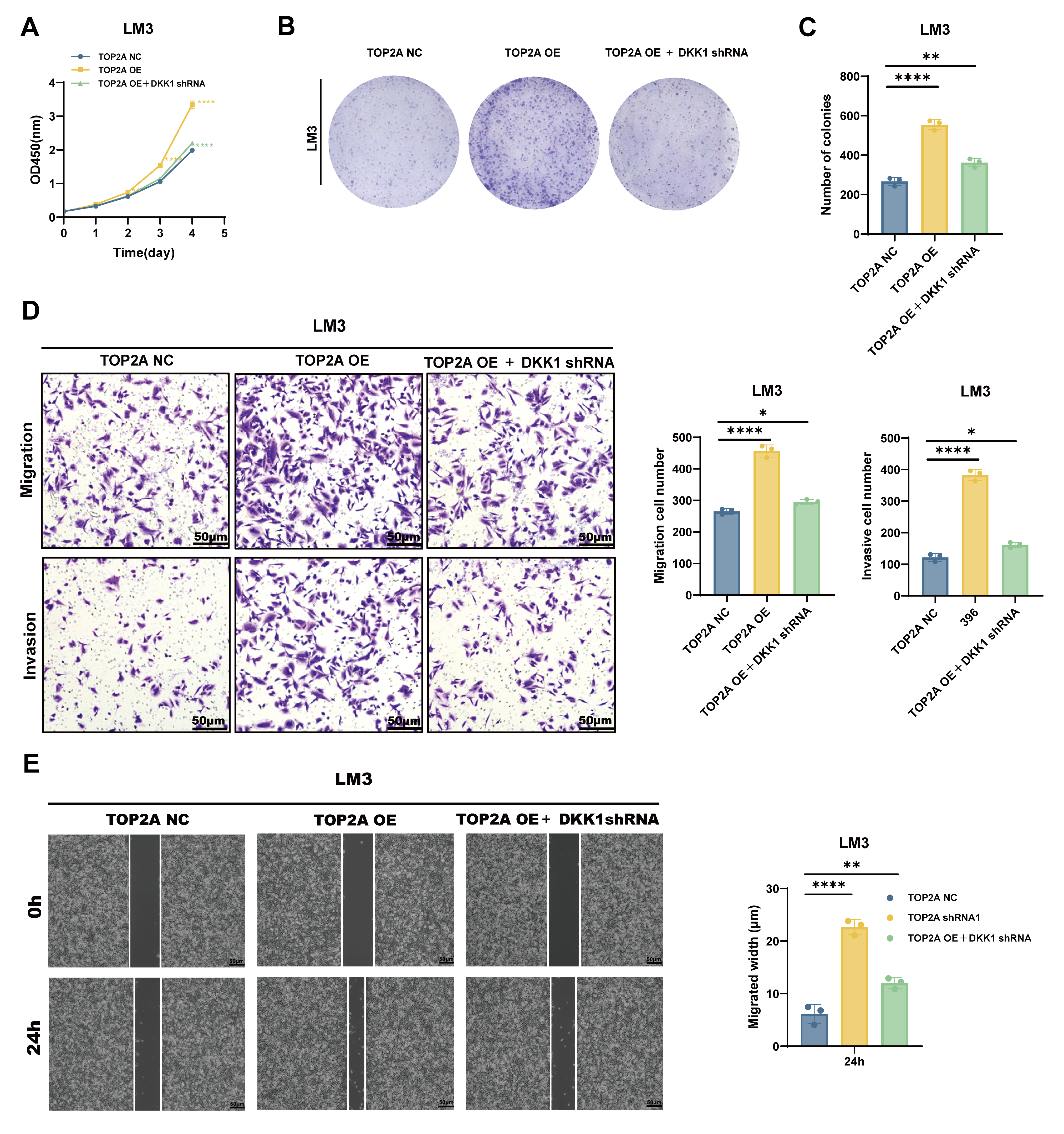

Supplement: Supplementary Figure 6 — DKK1 is required for TOP2A-induced malignant phenotypes in HCCLM3 cells. (A) CCK-8 assay showing the effect of TOP2A overexpression (TOP2A OE) and DKK1 knockdown (TOP2A OE + DKK1 shRNA) on the proliferation of HCCLM3 cells compared with control (TOP2A NC). (B, C) Colony formation assay demonstrating that TOP2A overexpression significantly enhances clonogenic ability, which is markedly attenuated by DKK1 knockdown. Quantification of colony numbers is shown in (C, D) Transwell migration and invasion assays showing that TOP2A overexpression promotes migratory and invasive capacities of HCCLM3 cells, while DKK1 knockdown significantly reverses these effects. Representative images are shown on the left, and quantitative analysis is shown on the right. Scale bar = 50 μm. (E) Wound healing assay indicating that TOP2A overexpression accelerates wound closure in HCCLM3 cells, whereas DKK1 knockdown suppresses this effect. Representative images at 0 h and 24 h are shown on the left, with quantitative analysis of migrated distance shown on the right. Data are presented as mean ± SD. Statistical significance was determined using appropriate tests (*P < 0.05, **P < 0.01, ***P < 0.001, ****P < 0.0001). [file Image6.tif]

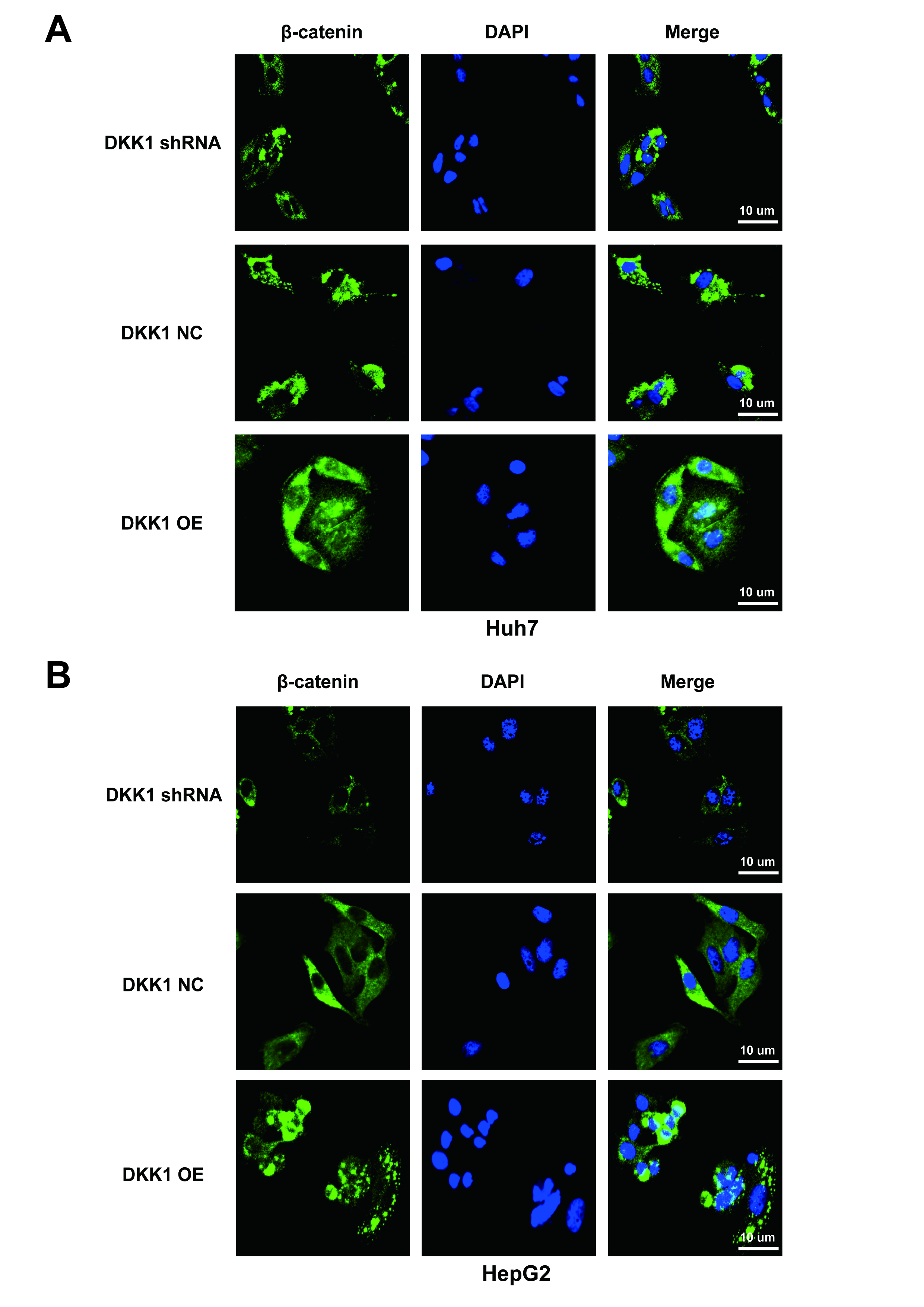

Supplement: Supplementary Figure 7 — DKK1 regulates β-catenin subcellular localization in HCC cells. (A) Huh7 cells and (B) HepG2 cells were subjected to DKK1 knockdown (DKK1 shRNA), negative control (DKK1 NC), or DKK1 overexpression (DKK1 OE). β-catenin was detected by immunofluorescence (green), and nuclei were counterstained with DAPI (blue). Merged images are shown in the right panels. Representative images indicate that modulation of DKK1 expression alters the distribution pattern of β-catenin in both cell lines. Scale bar, 10 μm. [file Image7.tif]

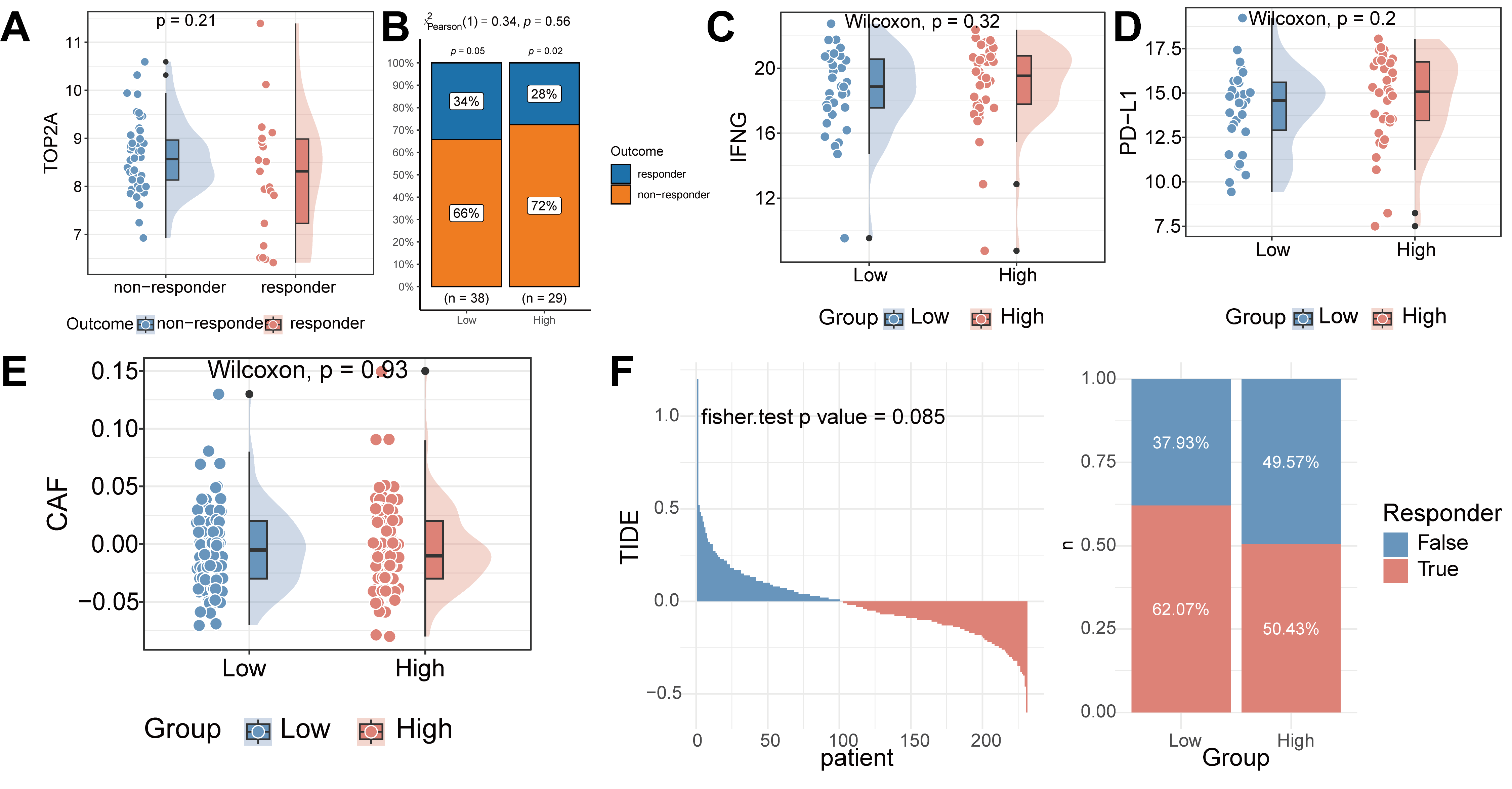

Supplement: Supplementary Figure 8 — Association of TOP2A expression with response to sorafenib and immunotherapy. (A) TOP2A expression levels in sorafenib responders versus non-responders within the GSE109211 dataset. (B) Proportion of sorafenib responders stratified by high and low TOP2A expression in the GSE109211 cohort. (C-E) Differences in immune-related scores—including IFNG, PD-L1 and CAFs—between patients with high and low TOP2A expression in the ICGC cohort. (F) Predicted response to immunotherapy between high and low TOP2A expression groups in the ICGC cohort, based on TIDE algorithm analysis. [file Image8.tif]
